# Single-cell lineage tracking analysis reveals that an established cell line comprises putative cancer stem cells and their heterogeneous progeny

**DOI:** 10.1038/srep23328

**Published:** 2016-03-22

**Authors:** Sachiko Sato, Ann Rancourt, Yukiko Sato, Masahiko S. Satoh

**Affiliations:** 1 Glycobiology and Bioimaging Laboratory of Research Center for Infectious Diseases, CHU de Québec, Faculty of Medicine, Laval University, 2705 Boulevard Laurier, Quebec, Québec G1V 4G2, Canada; 2 Laboratory of DNA Damage Responses and Bioimaging, CHU de Québec, Faculty of Medicine, Laval University, 2705 Boulevard Laurier, Québec, Québec G1V 4G2, Canada

## Abstract

Mammalian cell culture has been used in many biological studies on the assumption that a cell line comprises putatively homogeneous clonal cells, thereby sharing similar phenotypic features. This fundamental assumption has not yet been fully tested; therefore, we developed a method for the chronological analysis of individual HeLa cells. The analysis was performed by live cell imaging, tracking of every single cell recorded on imaging videos, and determining the fates of individual cells. We found that cell fate varied significantly, indicating that, in contrast to the assumption, the HeLa cell line is composed of highly heterogeneous cells. Furthermore, our results reveal that only a limited number of cells are immortal and renew themselves, giving rise to the remaining cells. These cells have reduced reproductive ability, creating a functionally heterogeneous cell population. Hence, the HeLa cell line is maintained by the limited number of immortal cells, which could be putative cancer stem cells.

Mammalian cell lines are often of clonal origin, therefore, it is assumed that they comprise putatively homogeneous clonal cells with similar phenotypic features. Indeed, many studies have been designed under the assumption. However, phenotypic characteristics of cultured cells change over time. Thus, cell lines come to comprise various phenotypically altered populations. The level of phenotypic homogeneity or heterogeneity of cultured cell populations has been determined by end point analyses, although these analyses reveal only the status of cells at specific times. Phenotypic changes of cultured cells occur chronologically, thus end point analyses cannot fully elucidate the level of phenotypic homogeneity or heterogeneity of cultured cell populations. The assumption has therefore remained untested.

Time-lapse cinematography can be used to obtain chronological data that list a sequence of events occurring in individual cultured cells. Previously, the analysis was carried out using a 16-mm film to make a live cell movie and cinematography to track cells individually^1^,^2^,^3^. However, analysis of individual cells by that method is laborious. In recent years, computer-controlled microscopes have been used for live cell imaging and cell tracking^4^,^5^,^6^,^7^. For instance, the nuclei of live cells are stained with a fluorescent dye and the cells are tracked using fluorescent imaging^8^, although this type of approach has the potential disadvantage that excitation of fluorescent dyes in cells causes phototoxicity, hindering accurate characterization of cells. In another approach, non-fluorescent imaging, for example, phase contrast or differential interference contrast (DIC) imaging, is used to visualize cells and live cell movies are used for cell tracking^6^,^9^,^10^, although the approach has never been used to analyze large numbers of cells. Thus, the characterization of cultured cells by obtaining the chronological data remains challenging.

To test the aforementioned assumption, we developed a method of chronological analysis with a DIC-based, single-cell lineage tracking method that can characterize every single cell recorded on live cell imaging videos. In this study, we used a cervical cancer cell line, HeLa S3 (HeLa), of which phenotype is likely to be predominant in the parental HeLa cell line^11^,^12^,^13^. Our study revealed unexpected characteristics of the HeLa cell line. The growth profile of individual HeLa cells varied significantly, and the majority of cells were mortal, in contrast to the general belief that HeLa cells are a cell line composed of immortal cells. Furthermore, only a small number of cells (3.2–6.1%) retained immortal growth ability, and gave rise to the remaining cell population. Our results thus do not support the assumption that the HeLa cell line comprises putatively homogeneous clonal cells. Instead, our results suggest that the HeLa cell line is maintained by the limited number of immortal cells, which could be putative cancer stem cells.

## Results

### Single-cell lineage tracking analysis

The microscope was designed to perform live cell imaging with an eight-well chambered coverglass for 100–200 h. Images were acquired using a 40× oil objective with a DIC filter, as DIC imaging was less disturbed by medium surface distortion compared with phase contrast imaging. We used a tungsten-halogen lamp as the light source. In each well, a two-dimensional image acquisition array (field of views: FOVs, Supplementary Fig. S1) was made to cover the area of interest. Images of each FOV were acquired every 10 min (Supplementary Movie S1 for the growth of HeLa cells on a microscope stage).

HeLa cells were plated at 3500 cells per well. Due to the physical nature of the wells, the plated cells were often unevenly spread and attached to the surface of the well. The cell density in a given area varied from 0 to 400 cells/mm^2^. We selected an area in which cell density was 180–220 cells/mm^2^.

**Figure 1:**
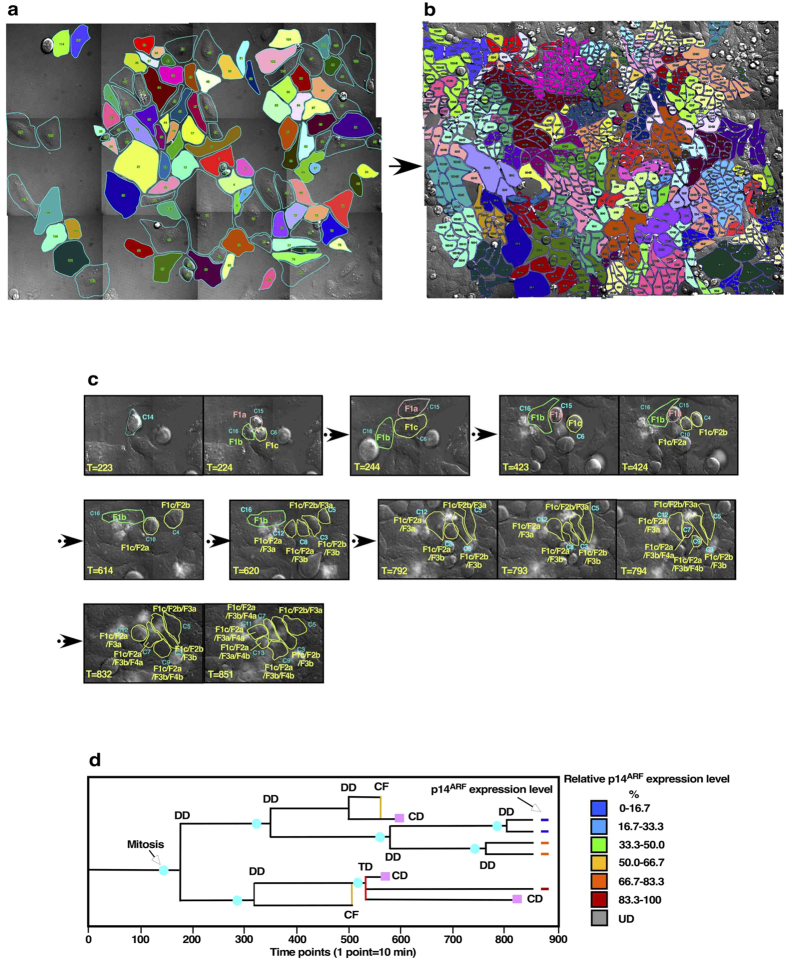
Outline of single-cell tracking analysis. (**a**,**b**) Representative images of Time point 1 (**a**) and Time point 850 (**b**) are shown. Images acquired from each FOV were stitched together and every cell (progenitor) recorded in the image of Time point 1 was numbered (**a**). Cell numbers were assigned all progeny cells produced from progenitors (**b**). A progenitor and its progeny cells were identified with a unique colors (**a**,**b**). (**c**) Still images shown in (**c**) illustrate how cell tracking was performed. The times and the types of all cellular events occur to progenitors and the progeny cells were recorded from Time point 1 till the end of the movie (typically Time point 850–950). For example, a cell (C14) identified at Time point 223 underwent TD at Time point 224, producing 3 progeny cells, C15 (F1a), C16 (F1b) and C6 (F1c). Then, progeny cells produced by the TD were tracked, time points, at which cellular events occurred, and types of events were recorded until the end of the movie. In this example, at Time point 424, C6 (F1c) underwent DD, producing 2 progeny cells, C10 (F1c/F2a) and C4 (F1c/F2b). Those progeny cells continue to proliferate and some of those underwent two more cell divisions. (**d**) An example of cell-lineage map is shown. Expression levels of p14^ARF^ in progeny cells were included in the map. UD: Undetectable. DD: Dipolar cell division. TD: Tripolar cell division. CF: Cell fusion. CD: Cell death.

To produce at least 100–150 cell lineage data, a panoramic image of FOVs was made (Fig. 1a). Every single cell in the image of Time point 1 was identified, and cell lineage numbers were assigned to the identified cells (Fig. 1a). We defined the cells, which were found in the image at Time point 1, as progenitors, and data that relate the progenitor to their progeny as cell lineage. Tracking of progenitors and their progeny was carried out from Time point 1 (Fig. 1a) to 850 (142 h, Fig. 1b) by visually following cells with a commercially available movie player. Still images illustrate how individual cells were followed, and time points at which cellular events occurred, were determined (Fig. 1c).

We categorized cellular events that were encountered during tracking (see Supplementary Fig. S2 for the full list of categorization). When a cell divided into two, three, four or five cells, these events were categorized as dipolar (DD), tripolar (TD, Supplementary Movie S2), tetrapolar (QD, Supplementary Movie S3) and pentapolar (PD, Supplementary Movie S4) cell division, respectively (Supplementary Fig. S2a–d). It has been reported that HeLa cells undergo TD at a frequency of 3–5% of total cell divisions, generating progeny with altered numbers of chromosomes^14^,^15^,^16^,^17^. We found that multipolar division (MD, which is the sum of TD, QD and PD) occurred in 5.8% of total cell divisions in the observation period of 130 h. If MD occurred stochastically, 1.1 ± 0.2 MD events per cell lineage would be expected. However, these events only occurred in 19.6% of cell lineages (Supplementary Fig. S3a). When the cell shape changed from flat to spherical, we considered that the cell entered mitosis. An event, where cells entered mitosis, but failed to divide and reattached to a cell culture surface, was defined as incomplete cell division (IP). We found various types of cell fusion (CF) events (Supplementary Fig. S2f–h). For example, CF occurred between non-mitotic cells (Supplementary Movie S5), between non-mitotic and mitotic cells (Supplementary Movie S6), and between mitotic cells. Those CF often occurred between progeny cells produced from same progenitor. CF occurred in 5.0% of total cell divisions, which was equivalent to 0.9 ± 0.2 events per cell lineage, if CF occurred stochastically. Similar to MD, however, CF occurred in only 31.6% of cell lineages and MD was often followed by CF (Supplementary Fig. S3a). Most cell death occurred after entering mitosis or the formation of a round mitotic-like cell (Supplementary Fig. S2i and Supplementary Movie S7).

Cell tracking data, including types of cellular events, were stored in the cell-lineage database to determine cell-lineage maps (Fig. 1d), which allowed us to characterize HeLa cells at the single-cell level in a chronological manner. For example, progenitors of any surviving progeny cells found at 142 h of culture (Fig. 1a, Progenitors and Fig. 1b, Progeny) can be identified by using the cell-lineage data. When indirect immunofluorescence was performed, expression levels of a protein in each cell can be included in the cell-lineage maps (Fig. 1d, p14^ARF^ expression level).

### Population doubling time of HeLa cells

**Figure 2:**
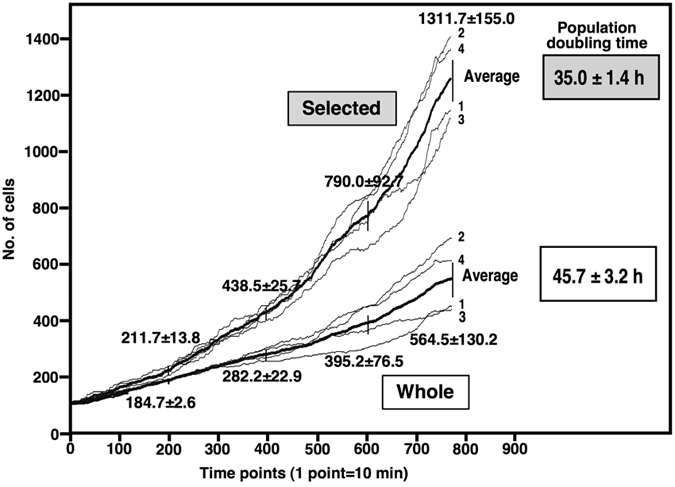
Population expansion curves. The number of cells at each time point was determined by using data stored in the cell-lineage database to draw cell population expansion curves. Four independent experiments (experiment number 1, 2, 3 and 4) were performed. The initial number of cells was normalized to 100 cells. The analyses were performed with data of all cells recorded in the cell-lineage database (Whole) or with data of selected group of cells (Selected), which belong to Group d–f (see Table 1). The average cell numbers were calculated (Average, bold line). The means and SDs of the number of cells at Time point 200, 400, 600 and 780 were shown and population doubling times for Whole and Selected were indicated. The average coefficient of variation was 9.45 (Whole) and 8.43% (Selected), suggesting that reproducible and quantitative cell biological analysis with long-term live cell imaging can be performed on a microscope stage.

A cell culture environment created on a microscope stage can be affected by various external factors, for example, the light used for visualization of cells and constant movement of the microscope stage during image acquisition. To test whether HeLa cells kept in an environment can grow reproducibly, four independent experiments for single-cell lineage tracking analysis were performed and cell population expansion rates were determined. The number of cells at every time point (every 10 min) was obtained using the cell-lineage database to draw cell population expansion curves (Fig. 2, Whole). Although some variations in the rates were observed after time point 400 (~66 h), there was no significant difference in the expansion rates among the four independent experiments. The average population doubling (Pop-Double) time was 45.7 ± 3.2 h, which was consistent with the doubling time measured for HeLa cells cultured in a CO_2_ incubator (42.6 h), and average coefficient of variation^18^,^19^ was 9.45% (Fig. 2, Whole), suggesting that reproducible and quantitative cell biological analysis with long-term live cell imaging can be performed on a microscope stage.

### Number of surviving progeny

Both the fate of each progenitor cell (see lineage maps in Supplementary Fig. S4) and the numbers of their surviving progeny (Supplementary Fig. S3b) varied significantly. We thus categorized progenitors into Groups a–f by the number of surviving progeny cells found at 130 h (Time point 780) of culture (Table 1). As many as 26.8% and 23.6% of progenitors produced no or no more than four surviving progeny cells, respectively (Table 1, Groups a and b). In contrast, 2.4% of progenitors produced ≥17 surviving progeny cells and the percentage of the progeny cells increased to 10.2% at 130 h (Table 1, Group f). The percentage of progenitors in Groups d–f was 25.9%, which increased to 61.1% at the observation period of 130 h. In contrast, the percentage of cells that belonged to Groups a and b (50.4% as progenitors) was reduced to 10.7%. These results indicate that reproductive ability of progenitors varied significantly and only <50% of progenitors were involved in the expansion of the HeLa cell population. Then, we performed a simulation of the growth of cells belonging to each group using a formula determined based on the growth curve of each group (Supplementary Fig. S5a–f). This simulation (Supplementary Fig. S5g–j) suggests that the percentage of cells that belonged to Group f reached 94.3% at 3333.3 h (Supplementary Fig. S5j) and thus the HeLa cell population could be replaced with progeny cells belonging to Group f after 138.9 days or 19.8 weeks of culture, if it assumes that the cells belonging to Group f retain its reproductive abilities.

### Re-determination of Pop-Double time of HeLa cells that belong to groups with higher reproductive ability

In conventional methods, cell Pop-Double time is determined under the assumption that most cells equally contribute to population expansion. However, our results suggest that <50% of progenitors contributed to population expansion. Thus, we recalculated Pop-Double time by selecting cell-lineage data related to Group d–f cells. The numbers of progenitors in Groups d–f and their surviving progeny cells at every time point were determined, population expansion curves were drawn, and the Pop-Double time was re-calculated (Fig. 2, Selected). The Pop-Double time was 35.0 ± 1.4 h (recalculated population doubling time: ReCal-Pop-Double), indicating that the Pop-Double time of HeLa cells (Groups d–f) was ~10 h shorter than the previously determined Pop-Double time (45.7 ± 3.2 h).

### Cell doubling time

**Figure 3:**
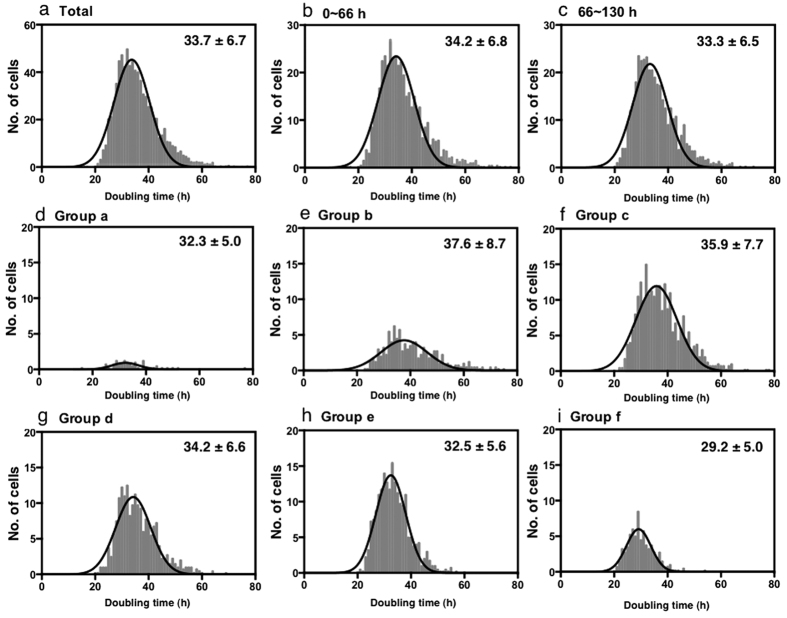
Cell doubling time. Cell doubling time of each cell was determined using data stored in the cell-lineage database. (**a**) All cells recorded in the cell lineage database. (**b**) Cells, which underwent cell division between 0–66 h. (**c**) Cells, which underwent cell division between 66–130 h. Cell doubling times of Group a–f cells (see Table 1 for categorization) are shown. (**d**) Cells of Group a. (**e**) Cells of Group b. (**f**) Cells of Group c. (**g**) Cells of Group d. (**h**) Cells of Group e. (**i**) Cells of Group f. Average cell doubling times and SDs, which were calculated using nonlinear regression-Gaussian equation (GraphPad Prism 6), are shown.

We next addressed whether the ReCal-Pop-Double time represented the absolute doubling time of HeLa cells. Cell doubling (Cell-Double) time at the single-cell level was determined by calculating the time required for each single cell to produce its daughter cells. The time when a cell was generated by cell division (Time 1) and the time when the same cell produced daughter cells (Time 2) were noted, and the Cell-Double time was calculated by subtracting Time 1 from Time 2. We performed the analysis using data stored in the cell-lineage database. The average Cell-Double time of HeLa cells was 33.7 ± 6.7 h (Fig. 3a and Supplementary Fig. S6a), which was close to the ReCal-Pop-Double time of 35.0 ± 1.4 h, indicating that the absolute doubling time of HeLa cells is 33–35 h.

Cell density reached 100% by 80 h of culture, and we then examined whether the cell density had any impact on the Cell-Double time. We divided the cells in the cell-lineage database into two groups; cells that divided between 0 and 66 h (Fig. 3b and Supplementary Fig. S6a) and between 66 and 130 h (Fig. 3c and Supplementary Fig. S6a). Both groups of cells showed similar average Cell-Double times (34.2 ± 6.8 and 33.2 ± 6.5 h). Thus, the density of cultured HeLa cells did not affect Cell-Double time, confirming that HeLa cells escaped from contact inhibition due to malignant transformation.

We then determined whether Cell-Double times differed among Groups a–f, which had different levels of reproductive ability (Fig. 3d–i and Supplementary Fig. S6b). Although majority of the cells in Group a did not proliferate, dividing cells in Group a had an average Cell-Double time of 32.3 h (Fig. 3d), suggesting that there was no significant prolongation of Cell-Double time in dividing cells of Group a, even though these progeny cells were unable to survive till 130 h of culture. Notably, the average Cell-Double times of cells, which belonged to the Groups with higher reproductive ability, were significantly shorter than in cells with lower reproduction ability [e.g. Group f (Fig. 3i) vs. Group b (Fig. 3e)(Student’s t-test, p < 0.00001), Supplementary Fig. S6b, and Supplementary Fig. S6c (correlation r = −0.93, p < 0.0001)], revealing an inverse correlation between reproductive ability and Cell-Double time.

### Reduction of reproductive ability

**Figure 4:**
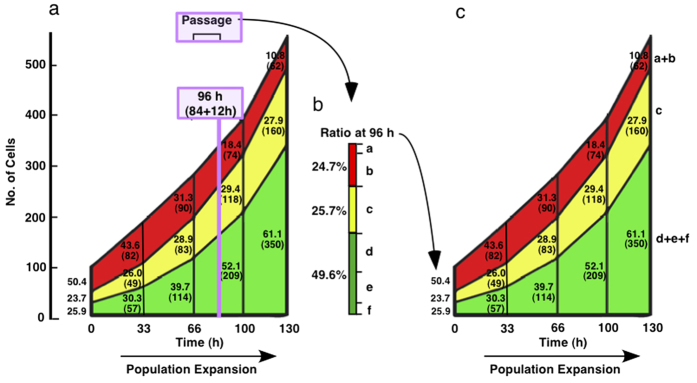
Change in the composition of HeLa cell culture in the observation period of 130 h. (**a**) Change in the composition of cells that belong to Group a–f (see Table 1) during the observation period of 130 h is shown. The total cell number at Time 1 is set as 100. The numbers at the left side of bars at 33, 66, 100 and 130 are the percentage of Group a+b, Group c and Group d–f, and the numbers in the parentheses is the number of cells. Passage of HeLa culture is generally performed 3–4 days (72–96 h) after cell plating (Passage). The line (light purple) indicates 96 h after cell plating. Because live cell imaging was started after 12 h of cell plating, 84 h of imaging is equivalent to 96 h after cell plating. (**b**) The composition of Group a–f cells at 96 h (84 h of imaging) is shown. (**c**) Frame (**c**) illustrates that HeLa cells show similar growth profile every after cell passage. The data shown in this frame is identical with that in frame (**a**).

The growth pattern of cells in Groups a–f during the observation period of 130 h is summarized in Fig. 4a. The percentage of Group a+b cells (indicated as the red region) reduced and Group d–f cells (the green region) increased over time. The percentage of Group d–f cells at 130 h of culture reached 61.1%. If those well-proliferating 61.1% of progeny cells continuously retained the growth ability of their progenitors, Pop-Doubling time would be expected to eventually approach the ReCal-Pop-Double time (35 h) after several cell passages. However, the Pop-Double time of HeLa cells was consistently ~46 h (Fig. 2, Whole) rather than 35 h, and such a change has not been noted during the maintenance of HeLa cell culture. We used HeLa cells from Passages 3, 5, 15 and 30 in four different experiments, and the Pop-Double times remained at ~46 h (Fig. 2, Whole). Thus, HeLa cells showed a similar growth profile after each cell passage (Fig. 4a,c). To understand the underlying mechanism by which HeLa cells show such a growth profile after each cell passage, we extracted single cell data at 96 h of culture from the cell-lineage database, as the passage of HeLa cells is generally performed between 72 and 96 h (Fig. 4a, Passage). The percentage of Group a–f cells at 96 h is shown in Fig. 4b. The total percentage of Group a+b cells was reduced to 24.7% from 50.4% and, the percentage of Group d–f cells was increased to 49.6% from 25.9%. When the composition of cells shown in Fig. 4b is put into a new culture, 49.6%, of cells are expected to produce ≥9 surviving progeny cells in the new culture (Group d–f), if those cells maintain their reproductive activity. However, the experimental data instead suggest that nearly half of the cells cannot produce ≥9 surviving progeny cells in the new culture, raising the possibility that they have lost their reproductive ability.

### Mortal and immortal HeLa cells

**Figure 5:**
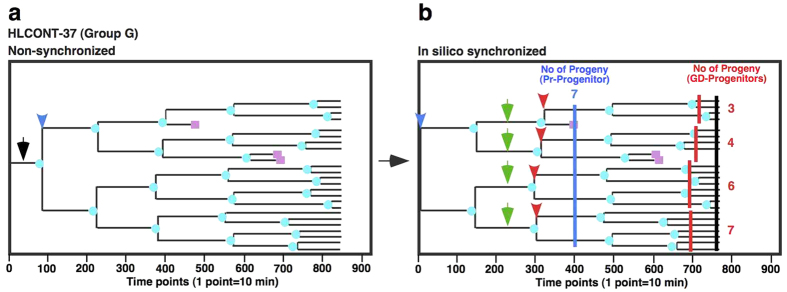
An outline of in depth analysis of cell-lineage database. (**a**,**b**) An example of in depth analysis of cell-lineage database is shown. (**a**) A progenitor cell (Pr-Progenitor, black arrow) of a cell lineage underwent the first cell division at the time point indicated by blue arrowhead. (**b**) When *in silico* synchronization of cell cycle was performed, the time when the Pr-Progenitor cell undergoes the first division is now defined as Time point 1 (blue arrowhead). Pr-progenitor cells were categorized by the number of surviving progeny cells found after 130 h of culture. Pr-progenitor cells produced 0, 1–4, 5–8, 9–12, 13–16, 17–20 and ≥21 surviving progeny were categorized as Group A, B, C, D, E, F and G, respectively. As the Pr-Progenitor cell produced 23 surviving progeny cells after the 130 h of culture (black line) from the first cell division, the Pr-Progenitor cell shown in (**b)** belongs to Group G and gave rise to 7 surviving progeny cells after 66 h of culture (blue line) from the first cell division. Its 4 granddaughter cells (GD-Progenitors) are indicated by green arrows and those cells undergo the first cell division at the time point indicated by red arrowheads. The GD-Progenitor cells gave rise to 3–7 surviving progeny cells after 66 h of culture (red line) from the first division.

**Figure 6:**
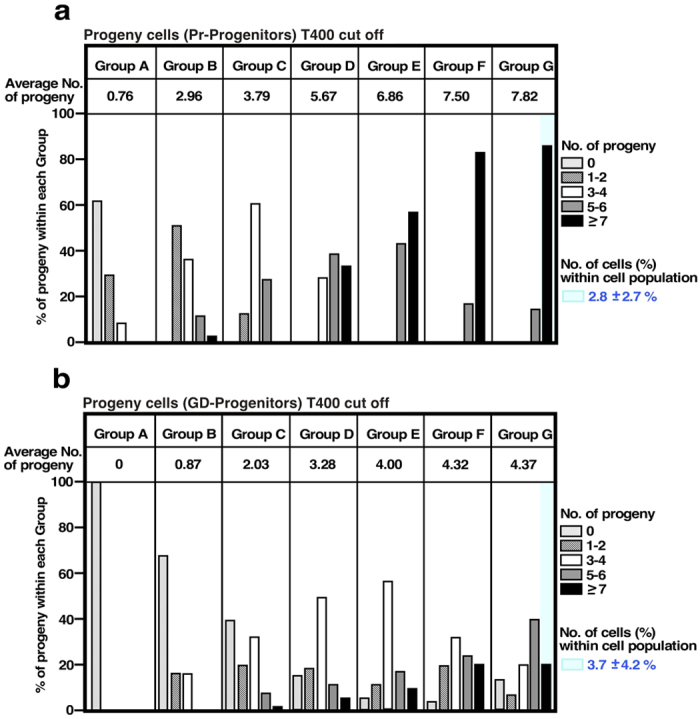
In depth analysis of cell-lineage database to identify mortal and immortal cells. (**a**) In depth analysis was performed on all cell lineages and the data were reassembled according to the groups (Group A–G) designated as Pr-Progenitor. The number of surviving progeny cells found after 66 h of culture from the first cell division of Pr-Progenitor cells was determined and results were arranged based on the number of surviving progeny. The Pr-Progenitor cell shown in Fig. 5b is an example cells that belongs to Group G and, as the cell produced 7 surviving progeny cells after 66 h of culture, the example cell was categorized as a class, which produces ≥7 progeny cells (black column). (**b**) The data were reassembled according to the groups (Group A–G) as in (**a**) The number of surviving progeny cells of GD-progenitor cells found after 66 h of culture from the first cell division was determined and results were arranged based on the number of surviving progeny cells. The GD-Progenitor cell shown in Fig. 5b is an example belongs Group G and, as the cells produced 4–8 surviving progeny cells after 66 h of culture, the example cells were categorized as classes, which produce 4 and ≥7 progeny cells (white and black column). (**a**,**b**) The numbers shown in each column are the average number of progeny cells. In Group G column, Pr-Progenitors or GD-Progenitors, which produced ≥7 surviving progeny cells, are highlighted by blue column. The percentages of those cells within the entire cell population were calculated and means and SD are shown (at the right side of blue box).

We performed a new in-depth data analysis using the cell-lineage database to address directly whether progeny cells retained or lost their reproductive ability, compared to their progenitors during expansion in culture, by performing *in silico* synchronization of the cell cycle. The time when a progenitor [Primary progenitor; Pr-Progenitor; Fig. 5a (non-synchronized) shows an example, black arrow] undergoes the first division (Fig. 5a, blue arrowhead) is now defined as Time point 1 [Fig. 5b (*in silico* synchronized), blue arrowhead]. The number of its surviving progeny cells at 130 h (Fig. 5b, Time point 780, black line) from the first cell division (Fig. 5b, Time point 1, blue arrowhead) was determined. After applying this *in silico* synchronization to all Pr-Progenitors, we re-categorized the Pr-progenitors based on the numbers of their progeny cells found 130 h after the first cell division of Pr-Progenitors (Fig. 5 legend). To distinguish these groups from those categorized with non-synchronized progenitors, re-categorized groups were designated as Groups A–G. We then compared the reproductive abilities of both Pr-Progenitors and their grand-daughter cells (Fig. 5b, green arrows). Analysis of a Pr-Progenitor in Group G is shown in Fig. 5b (see HLCONT-37 in Supplementary Fig. S4). The Pr-Progenitor gave rise to 7 surviving progeny cells after 66 h of culture (Fig. 5b, blue line, Time point 400, 66 h). To study whether its grand-daughter cells (GD-Progenitors, Fig. 5b, green arrows) retained similar reproductive ability as the Pr-Progenitors, the number of surviving progeny cells produced by the GD-Progenitors after 66 h (Fig. 5b, red lines) of their first cell division (Fig. 5b, red arrowheads) was also determined. In this example, there were 3 to 7 progeny cells of the GD-Progenitors (Fig. 5b, numbers at the end of cell-lineage maps) and, only one GD-Progenitors retained their reproductive ability. The analysis was performed on all cell lineages and the data were reassembled according to the groups designated as Pr-Progenitor. The numbers of progeny cells produced by Pr-Progenitor (Fig. 6a) and GD-Progenitor (Fig. 6b) cells were determined. It should be noted that the analysis was not affected by the difference in cell culture density since Cell-Double time of HeLa cells was not reduced by cell density (Fig. 3b,c). If GD-Progenitors retain similar growth ability to their Pr-Progenitors, it would be expected that both cell types would produce similar numbers of progeny cells. However, our analysis revealed a clear tendency that GD-Progenitors consistently had reduced reproductive ability compared to Pr-progenitors (Fig. 6a,b; average number of progeny cells is shown). Similar results were obtained when the number of surviving progeny cells produced by the GD-Progenitors 58 h after their first cell division was determined (Supplementary Fig. S7a,b), again suggesting that HeLa cells become mortal during their expansion.

If the majority of HeLa cells were mortal, culture would likely cease. However, the immortality of HeLa cells has been maintained for >60 years, indicating the presence of cells that have retained immortality. Candidate immortal cells are expected to produce two types of progeny cells; one has similar and the other has reduced reproductive ability. Cells that have such characteristics could belong to Group G. The GD-Progenitors, which showed lower reproductive ability than Pr-Progenitors, were produced from Group G cells (Fig. 6a,b), while some of the GD-Progenitors produced a similar number of progeny cells as Pr-Progenitors did (Fig. 6a,b, blue box). Furthermore, if some HeLa cells played a role in maintaining cell immortality, we would expect a constant number of immortal cells in the HeLa cell population. Thus, we determined the percentage of Group G cells, which produced ≥7 surviving progeny cells (Fig. 6a,b, blue box). In the case of Pr-progenitors, the percentage of such progeny cells was 2.8 ± 2.7% of the total cell population (Fig. 6a). The percentage of GD-Progenitors that produced ≥7 surviving progeny cells was similarly 3.7 ± 4.2% (Fig. 6b). We made a similar determination with Group F and G cells and the percentages of Pr-progenitors and of GD-Progenitors that produced ≥7 surviving progeny cells were 5.1 ± 4.1% and 7.1 ± 6.8%, respectively, indicating that 2.8%–3.7% (Group G cells: ~3.2%) or 5.1–7.1% (Group F and G cells: ~6.1%) of HeLa cells retained their higher reproductive ability and immortality. Results shown in Supplementary Fig. S8a suggest that >90% of growing cells found in HeLa cell populations could be cells that are losing immortality.

To address the reason why most cells fail to retain their reproductive ability, we also analyzed the relationship between abnormal cellular events (MD and CF), cell death and the number of progeny cells produced by GD-Progenitors. Most MD and CF events were found in cell lineages that were committed to cell growth termination (Supplementary Fig. S7c). Interestingly, 39% of cell death occurred following MD or CF (Supplementary Fig. S8b: 20.8% for MD and 18.2% for CF) and 84.7% of progeny produced by MD underwent cell death, consistent with the observations made by Ganem *et al.*^14^. Furthermore, cell death occurred more frequently in the same groups of cells (Supplementary Fig. S7d), suggesting that MD, CF and cell death occur in cells with reduced reproductive ability, leading to the removal of these cells from the population.

### Dynamic change in HeLa cell phenotype

To study whether phenotypic changes occurred during propagation of HeLa cells, we combined single-cell tracking analysis with indirect immunofluorescence. We chose p14^ARF^, which is a tumor suppressor that is highly expressed in HeLa cells^20^,^21^, as the protein is concentrated in nucleoli, allowing quantitative analysis in each cell. At the end of live cell imaging, the multiwell chamber was removed from a microscope stage, cells were fixed, and p14^ARF^ was stained. The chamber was put back to the microscope stage, identical cells that had been monitored were identified, and both DIC and fluorescent imaging were performed (Supplementary Fig. S9). Expression of p14^ARF^ was determined and the results were incorporated into cell-lineage maps (examples are shown in Fig. 1d and Supplementary Fig. S4). Incorporating the results of immunofluorescence staining with cell-lineage maps provides a method to verify errors in quantitation, since it can be adjusted by comparison of the expression level of p14^ARF^ of a cell with its siblings. For example, a group of siblings in Supplementary Fig. S10a (light blue shade, right bar, 36) consistently showed a lower level of p14^ARF^ expression, allowing us to verify the results of quantitation. We traced back the cell lineage to find a cell division that altered the expression level of p14^ARF^. In the case of HLCONT-21, an alteration occurred at a DD (indicated by the arrow, Supplementary Fig. S10a). We performed similar analysis with HLCONT-76 (Supplementary Fig. S10b) and HLCONT-37 (Supplementary Fig. S10c). These results obtained by the analysis of p14^ARF^ expression indicate that progeny cells that carry an altered phenotype are produced during or after cell division, resulting in a dynamically changed phenotype of HeLa cells, including ones, which retain immortality.

## Discussion

**Figure 7:**
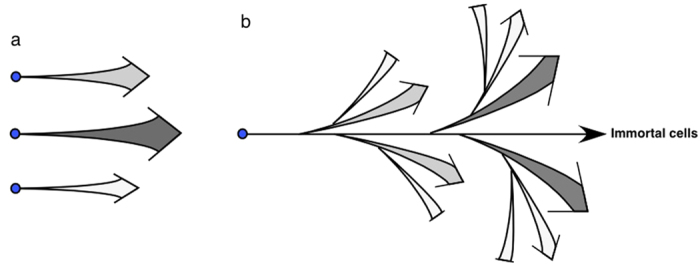
A model for the growth profile of HeLa cells. (**a**) A classical model: Illustration for the assumption that cell lines comprise putatively homogeneous clonal cells. Although there are some variations in growth rate among cells and in phenotypic characteristics (illustrated by different gray colors), these cells are clonally expand every after cell passage. (**b**) A model proposed based on the analysis with the cell-lineage database: Small number of immortal cells play a role in maintaining HeLa cell line and produce mortal HeLa cell population. Mortal cells have reduced reproductive ability (groups with arrow) and eventually terminate their growth (block ends). Phenotypic changes also occur during HeLa cell growth (illustrated by different gray colors).

In this study, we tested the assumption that cell lines comprise putatively homogeneous clonal cells with similar phenotypic features using single-cell lineage analysis of HeLa cells. Although the phenotypic features of cultured cells change over time, the growth profile of cultured cells has been considered as the model illustrated in Fig. 7a. The majority of cells grow at similar rates because they are considered to be putatively homogeneous clonal cells. Even though some phenotypic changes accumulate in some cells, the proportion of those cells in the population could remain constant after every cell passage. Results of various cell biological studies have been interpreted based on this putative model, which have not been validated till now. In our study with HeLa cells, a chronological approach using a single-cell lineage database did not support the above model and the previous assumption. Instead, the analysis suggests that the HeLa cell line comprises highly heterogeneous cells; most of which are mortal cells with various levels of reproductive ability. In-depth analysis revealed that only a small number of cells retained their immortality (3.2–6.1%, Fig. 6) and the remaining cell population was likely derived from these immortal cells (Fig. 7b). These cells eventually become mortal, as the ability of cells to produce progeny is progressively reduced during a short period of cell expansion, leading to growth termination. In addition, the phenotypes of immortal cells changed during their expansion (Supplementary Fig. S10), suggesting that the characteristics of HeLa cells also change over time. In summary, in contrast to the general belief that HeLa cells are a cell line composed of immortal cells (Fig. 7a), only a limited number of cells (3.2–6.1%) are immortal cells with the ability to self-renew, giving rise to the functionally heterogeneous cell populations, which are later changed to cells with reduced or no reproductive ability (Fig. 7b).

Although the aforementioned assumption has often been made, the presence of heterogeneous reproductive ability in various established cell lines has also been realized for more than several decades. For example, single cells plated onto a culture dish formed different sizes of colonies and cells comprising original HeLa and HeLa S3 cell lines are also known to form various sizes of colonies^11^,^12^,^13^, suggesting that those cell lines are composed of cells with different reproductive abilities. Those observations have been contributed to develop a conceptual understanding in heterogeneity of cultured cells. However, as phenotypic changes of cultured cells occur chronologically, end point analyses (e.g. colony formation assay) cannot fully elucidate the nature of heterogeneity of cultured cell populations. We demonstrated here that a direct visual proof of the presence of heterogeneity in HeLa cells can be obtained using a chronological analysis, i.e. single-cell lineage tracking analysis.

The precise characteristics of cells that retain higher reproductive ability and immortality are not yet known. Because HeLa cells are derived from cervical cancer tissue^12^ and the cells that retain higher reproductive ability and immortality could produce both immortal and heterogeneous mortal progeny (Fig. 7b), we tentatively define those cells as putative cancer stem cells, as cancer stem cells have the ability to self-renew and give rise to heterogeneous progeny^22^. Putative cancer stem cells may be similar to cancer stem cells defined using various markers^22^, or the putative cancer stem cells may be a distinct type of cell that cannot be defined by the existing concept of cancer stem cells. To characterize putative cancer stem cells or rare cell population, which can be found in established cultured cells, it would require to combine single-cell lineage analysis with other end point analyses, for example, indirect immunofluorescence, single-cell mRNA quantitation and label-retaining assay, to perform quantitative analysis of cancer stem cell and/or asymmetric cell division markers. Although it still needs to develop a new technique to combine those analyses, *in silico* analysis of cell lineage data associated with data obtained using end point analyses would became a powerful research method, as the analysis will allow to accurately determine the status of cells, e.g. by synchronization of cells without exposing cells to low serum medium or simulating the expansion of cells of interest using the cell lineage database and a mathematical model.

Finally, the unexpected properties of HeLa cell culture revealed by our new approach led us to consider the process by which HeLa cell culture was established. Indeed, it is plausible that HeLa cell culture was successfully established because the original culture contained putative stem cells for cervical cancer. It is also conceivable that, during the cloning of HeLa cells, stem-cell-like cells present in cultured HeLa cells were isolated, as the cells could form larger colonies than mortal cells could. The HeLa cell line is one of the most widely used cell lines in the field of biomedical research. We thus believe that our study provides a new research direction using a system with HeLa cell lines, as the immortal cells that represent 3.2–6.1% of total cell population are likely to be true target for understanding the spectrum of cell behaviors, for evaluating the effect of anti-cancer drugs and for investigating the mechanism of immortal growth. We should also note that the assumption that cell lines comprise putatively homogeneous clonal cells with similar phenotypic features should be vigorously tested in other established human cell lines, as other cultured cells may grow in a similar manner to HeLa cells. Single-cell lineage tracking analysis that we developed is, however, still a laborious process. Currently, various cell tracking software and methods are being developed^23^,^24^,^25^. Although the development of such software is still challenging^23^, automated single-cell lineage tracking analysis combined with end point analyses will be a powerful laboratory tools for future cell biology research.

## Methods

### Cells

HeLa S3 cells were purchased from ATCC and cultured in DMEM containing 10% fetal bovine serum in a humidified 5% CO_2_. Passage of HeLa S3 cells was performed every 3 or 4 days and plated HeLa S3 cells at the 1 to 10 ratio. To plate cells onto a coverglass Lab-Tek 8 well chamber, 50 μl of HeLa S3 suspension containing 3500 cells were placed at the center of each well and left until cells attached to the coverglass surface. Then, 0.75 ml of culture medium was added to each well. If a 0.75 ml cell suspension, instead of 50 μl, was added to the well, cells tended to be concentrated at the wall. Thus, our method gave a better result to evenly spread cells in a given area, although some local variation of cell concentration was still observed. Cells were used for live cell imaging 12 hrs after the plating.

### Microscope and long-term live cell imaging

Quorum WaveFX Spinning Disc Confocal System (Quorum Technologies Inc., Canada) with Leica microscope controlled by Volocity v4.0 was employed for long-term live cell imaging. To eliminate any risk of phototoxicity, DIC images were taken through HCX PL APO 40x oil objectives (NA = 1.25) by using a Halogen lamp as a light source (UV light from the lamp was removed nearly to 100% by DIC prism filters). Cells grown on a coverglass Lab-Tek 8 well chamber were then placed on a microscope stage and were cultured using an environmental chamber at 37 °C with 7.5% humidified CO_2_ (Pathology Devices Inc, MD). XY positions of FOVs were then registered using Volocity v4.0. DIC images were captured every 10 min from +10 to −10 μm of the focal plan at every 1 μm using piezo focus drive. Exposure time was normally 34 msec. and, every 24 hrs, culture medium was replaced with fresh medium. In a typical experiment, the growth of HeLa cells under the condition was ~90% of that in CO_2_ incubator, e.g. 0.53 ± 0.10 × 10^5^ and 0.59 ± 0.05 × 10^5^ cells/well in the environmental chamber and in CO_2_ incubator, respectively, after 120 hrs of culture.

### Cell movie making

To make time-lapse videos, focused images were selected from 21 *z*-plane image files. Volocity image files were used to select the optimal focal plane for each FOV. After the selection, files containing focused image were assembled into movie files using QuickTime player Pro. If image quality was not optimal for cell tracking, contrast was adjusted using a batch processing function of Photoshop v7.0.

### Cell Tracking

Cell tracking was carried out manually. Panoramic views of Time point 1 were prepared and cell lineage numbers were assigned. After assigning cell lineage number, cells were tracked using QuickTime Player Pro. We recorded the times of mitosis, cell division, cell fusion and cell death. Most of the manual tracking was confirmed once by rewinding the movie. To ensure the accuracy of cell tracking, a panoramic image of the last time point of imaging was also used. The numbers of each progeny cell were noted on the image and when the number was assigned to a cell, or when cells with no progeny were found, single-cell lineage data were verified. To draw cell lineage maps and process data, Excel Visual Basic Macro programs were written.

### Indirect immunofluorescence

After long-term live cell imaging, a coverglass Lab-Tek 8 well chamber was removed from the microscope stage. Then, cells were fixed with 0.5% paraformaldehyde for 15 min at room temperature. After washing with phosphate-buffered saline (PBS) containing 0.1% Triton-X 100 and 0.1% bovine serum albumin (PBS-TB), cells were incubated with anti-p14^ARF^ antibody (2000 fold dilution, Abcam) in PBS-TB for 2 h at room temperature. Cells were washed with PBS containing Triton–X 100 (PBS-T) followed by incubation with Alexa Fluor 488 goat anti-mouse antibody (1000 fold dilution). The coverglass Lab-Tek 8 well chamber was placed back on the microscope stage. XY position of each FOV was readjusted to account for the 5 to 10 μm shift in cell position that often occurred during this process. Then, DIC and fluorescence images were acquired. The area of the nucleus of each cell was individually marked by using Canvas X and the sum of the value of each pixel within the marked area was determined. Relative signal intensity was calculated and the data was incorporated into cell-lineage maps.

## Additional Information

**How to cite this article**: Sato, S. *et al.* Single-cell lineage tracking analysis reveals that an established cell line comprises putative cancer stem cells and their heterogeneous progeny. *Sci. Rep.* **6**, 23328; doi: 10.1038/srep23328 (2016).

## Supplementary Material

Supplementary Information

Supplementary Movie S1

Supplementary Movie S2

Supplementary Movie S3

Supplementary Movie S4

Supplementary Movie S5

Supplementary Movie S6

Supplementary Movie S7

## Figures and Tables

**Table 1:** The number of progeny cells produced from a progenitor.

The number of surviving progeny cells	Progenitors (Time point 1, 0 h)			Progeny cells (Time point 780, 130 h)		
	The number of progenitors^1^	% Population		The number of progeny cells^1^	% Population	
Group a: 0	26.8 ± 3.9	26.8	50.4	0	0	10.7
Group b: 1–4	23.6 ± 6.2	23.6		61.8 ± 18.2	10.7	
Group c: 5–8	23.7 ± 5.2	23.7	23.7	160.3 ± 34.9	28.0	28.0
Group d: 9–12	14.5 ± 1.9	14.5	25.9	156.7 ± 26.3	27.3	61.3
Group e: 13–16	9.0 ± 4.4	9.0		136.1 ± 67.0	23.8	
Group f: ≥17	2.4 ± 3.7	2.4		57.5 ± 47	10.2	
Total	100.0	100	100	572.5	100	100

The table shows the numbers and percentages of progenitors (Time point 1, 0 h) and their surviving progeny cells found at Time point 780, 130 h).

^1^The indicated numbers are the means and SDs of data from 4 independent experiments.
