# Supplementary material for: Single-cell lineage tracking analysis reveals that an established cell line comprises putative cancer stem cells and their heterogeneous progeny
Source: Sci Rep. 2016 Mar 22;6:23328. doi: 10.1038/srep23328 (PMC4802345; doi:10.1038/srep23328)
Supplement: Supplementary Information [file srep23328-s1.pdf]

Supplementary information

Single-cell lineage tracking analysis reveals that an established cell line comprises putative cancer stem cells and their heterogeneous progeny

Sachiko Sato[1], Ann Rancourt[1, 2], Yukiko Sato[1,3], and Masahiko S. Satoh[2]*

[1]Glycobiology and Bioimaging Laboratory of Research Center for Infectious Diseases, and [2]Laboratory of DNA Damage Responses and Bioimaging, CHU de Québec, Faculty of Medicine, Laval University, 2705 Boulevard Laurier, Quebec, Quebec G1V 4G2, Canada

[3] Present address: Department of Physiology, McGill University, Montreal, Canada

*All correspondence should be addressed to Masahiko S. Satoh

Tel.: 1-418-525-4444 ext. 47340

e-mail: Masahiko.sato@crchul.ulaval.ca

Titles and Legends for Supplementary Figures

Supplementary Figure S1 Microscope setting for single-cell lineage tracking analysis
An 8-well chambered coverglass was placed on a microscope stage. DIC images were acquired using a 40x oil objective every 10 min. In each well, a two dimensional image acquisition array (filed of views: FOVs) was made to cover the area of interest. This figure illustrates an example of the movement of microscope stage. For example, the stage moves from FOV1 of well 1 (W1) to FOV15 of well 8 (W8).

Supplementary Figure S2 A Full list of categorized cellular events
A full list of categorized cellular events is shown. **a**. DD: Dipolar cell division. **b**. TD: Tripolar cell division. **c**. QD: Tetrapolar cell division. **d**. PD: Pentapolar cell division. **e**. IP: Incomplete cell division. **f**. Cell fusion between non-mitotic cells. **g**. Cell fusion between mitotic and non-mitotic cells. **h**. Cell fusion between mitotic cells. **i**. CD1: Cell death occurred after entering mitosis. **j**. CD2: Cell death occurred in the non-mitotic phase.

Supplementary Figure S3 Characteristics of progenitor cells
a. Progenitor cells, of which progeny underwent MD, CF and MD+CF during the observation period (Time point 1~780), are indicated. **b.** Progenitors were categorized by the number of their surviving progeny cells at Time point 780.

Supplementary Figure S4 Cell lineage maps
Examples of cell lineage maps are shown. The ID of this series of analysis is HELACONT. Watercolor square: Mitosis. Pink square: Cell death. Green square: Incomplete cell division. Black line: Cell fusion. Red Line: Multipolar cell division. Expression levels of p14^{ARF} in each progeny are also shown.

Supplementary Figure S5 Simulation of the growth of cells belonging to Group b-f
Growth of HeLa cells belonging to Group a-f was determined (**a**). Then, growth curve fittings (Prism 6, Equation: exponential growth) were performed. Curve fitting: Group b (**b**), Group c (**c**), Group d (**d**), Group e (**e**) and Group f (**f**)). The growth of cells belonging to Group b-f was simulated. Simulation: 0-20,000 min (**g**), 0-40,000 min (**h**), 0-100,000 min (**i**) and 0-200,000 min (**j**).

Supplementary Figure S6 Analysis of cell doubling time
Cell doubling times of individual cells were determined and plotted. **a**. All cells recorded in the cell-lineage database, cells that divided between 0-66 h and cells that divided between 66-130 h. **b.** Group a–f cells (see Table 1 for categorization). **c.** Correlation between the doubling time of individual cells and the number of surviving progeny at 130 h was determined. Prism 6 was used to calculate r and p values. Means and SDs are shown.

Supplementary Figure S7 In depth analysis of cell-lineage database to identify mortal and immortal cells using 58 h after the first cell division of Pr-Progenitors and GD- progenitors, and to analyze cells that underwent MD, CF and/or cell death
In silico synchronization analysis of cell cycle was performed and data was arranged as described in the legend of Fig. 5. **a.** The number of surviving progeny cells found after 58 h of culture from the first cell division of Pr-Progenitor cells was determined and results were arranged based on the number of surviving progeny. **b**. The data were reassembled according to the groups (Group A–G) as in **a**. The number of surviving progeny cells of GD-progenitor cells found after 58 h of culture from the first cell division was determined and results were arranged based on the number of surviving progeny cells. **a** and **b.** The numbers shown in each column are the average number of progeny cells. In Group G

column, Pr-Progenitors or GD-Progenitors, which produced ≥ 7 surviving progeny cells, are highlighted by blue column. The percentages of those cells within the entire cell population were calculated. Means and SD are shown (at the right side of blue box).
c. The number of Pr-Progenitor and/or their progeny cells, which underwent MD and/or CF in the period of 66 h was determined. **d.** The number of Pr-Progenitor and/or their progeny cells, which underwent cell death in the period of 66 h was determined.

Supplementary Figure S8 The analysis of cells, which retain reproductive ability, and of cellular events that occurred prior to cell death
a. The number of Group a-e and Group f cells at each time point was determined. Cells, which retained reproductive ability, were selected (cells that were capable to undergo DD). The percentages of Group a-e and Group f are shown. **b.** The number of cellular events occurred prior to cell death was determined. DD: Dipolar cell division. MD: Multipolar cell division. CF: Cell fusion. M: Mitosis.

Supplementary Figure S9 Single-cell indirect immunofluorescence
After performing live cell imaging, cells were fixed and $p14^{ARF}$ was stained using indirect immunofluorescence. Then, DIC (**a**) and fluorescent (**b**) images of cells were acquired. The nucleus of each cell was identified (**a**) and the expression levels of $p14^{ARF}$ in each nucleus were determined (**b**).

Supplementary Figure S10 Change in expression of $p14^{ARF}$ in HeLa S3 cells
The expression levels of $p14^{ARF}$ in progeny are indicated at the end of lineage lines (color coded bars). The values at the right side of the bars represent an average of relative expression level of $p14^{ARF}$. The white arrow indicates a DD (dipolar cell division) where the change in expression of $p14^{ARF}$ would occur. HLCONT-21 (**a**), HLCONT-76 (**b**) and HLCONT-37 (**c**) were analyzed.

Supplementary Figure S1

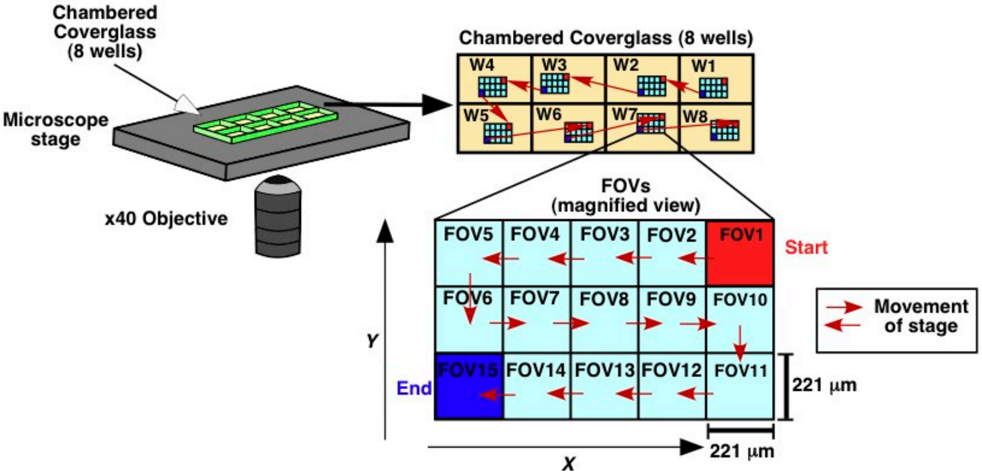

Supplementary Figure S2

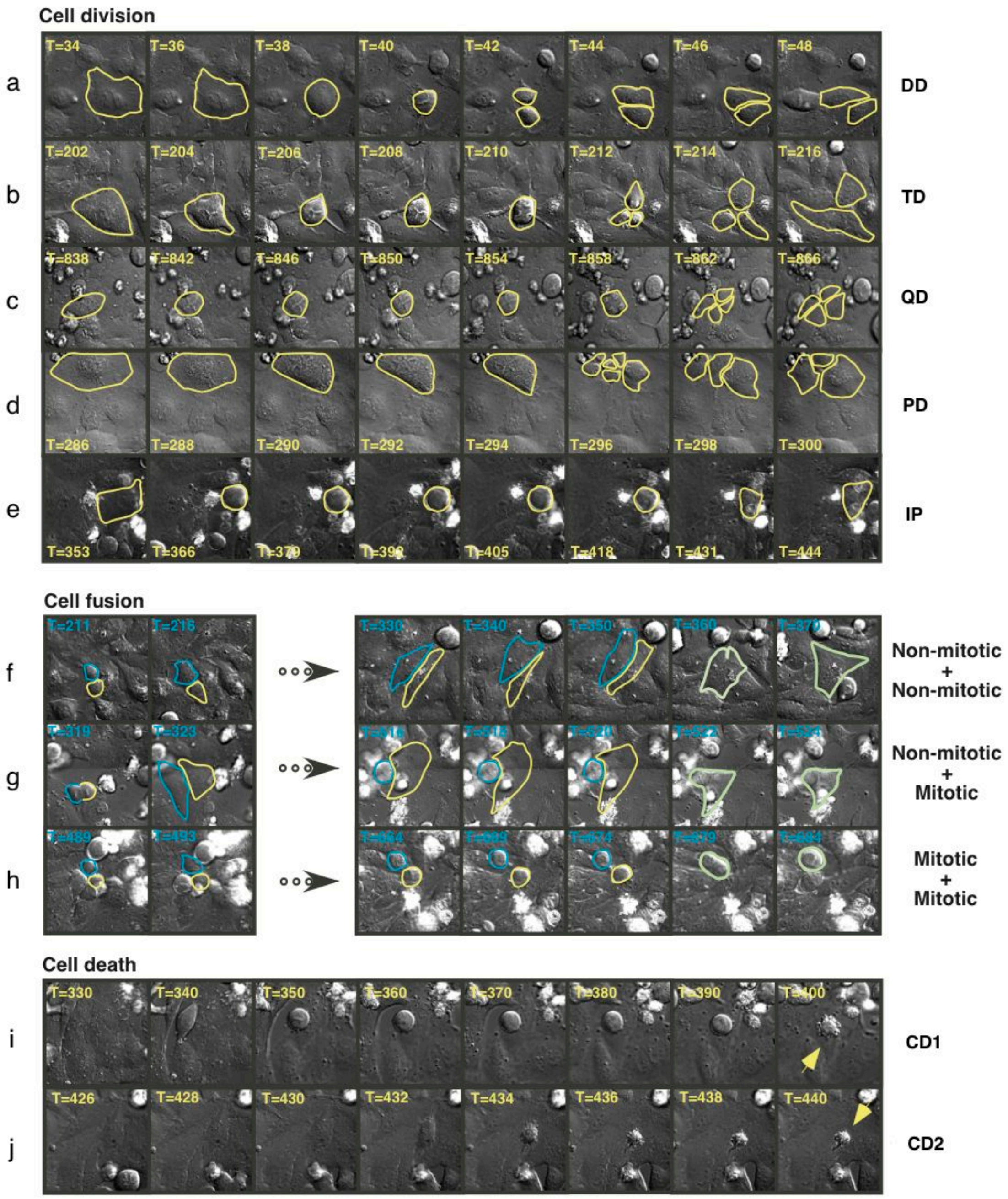

Supplementary Figure S3

a

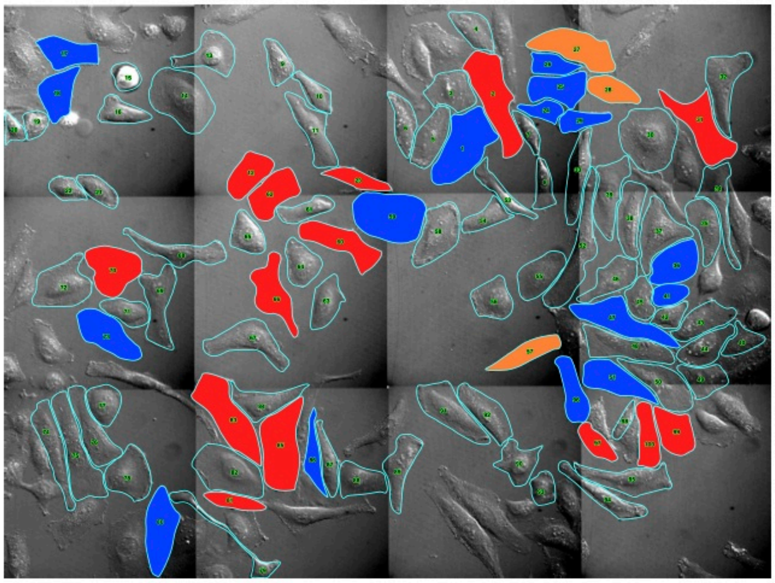

b

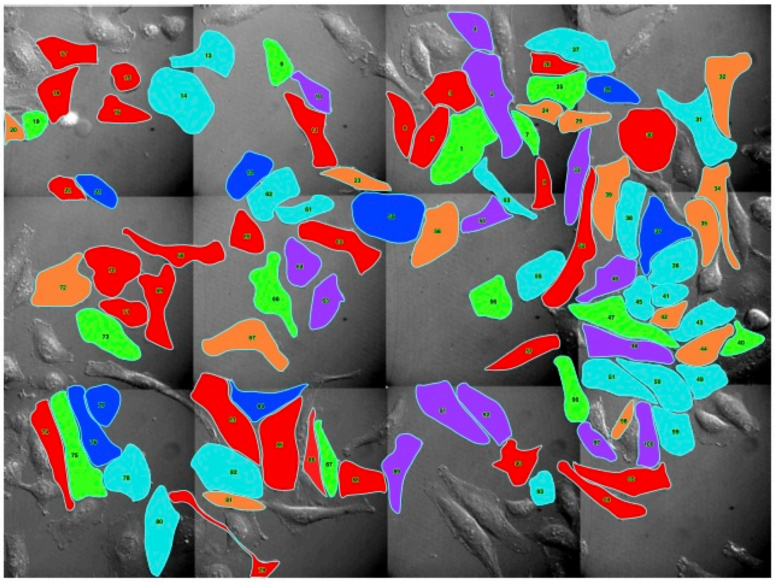

Supplementary Figure S4

Data Set 5 (HeLa Control R2), Lineage map 1-8

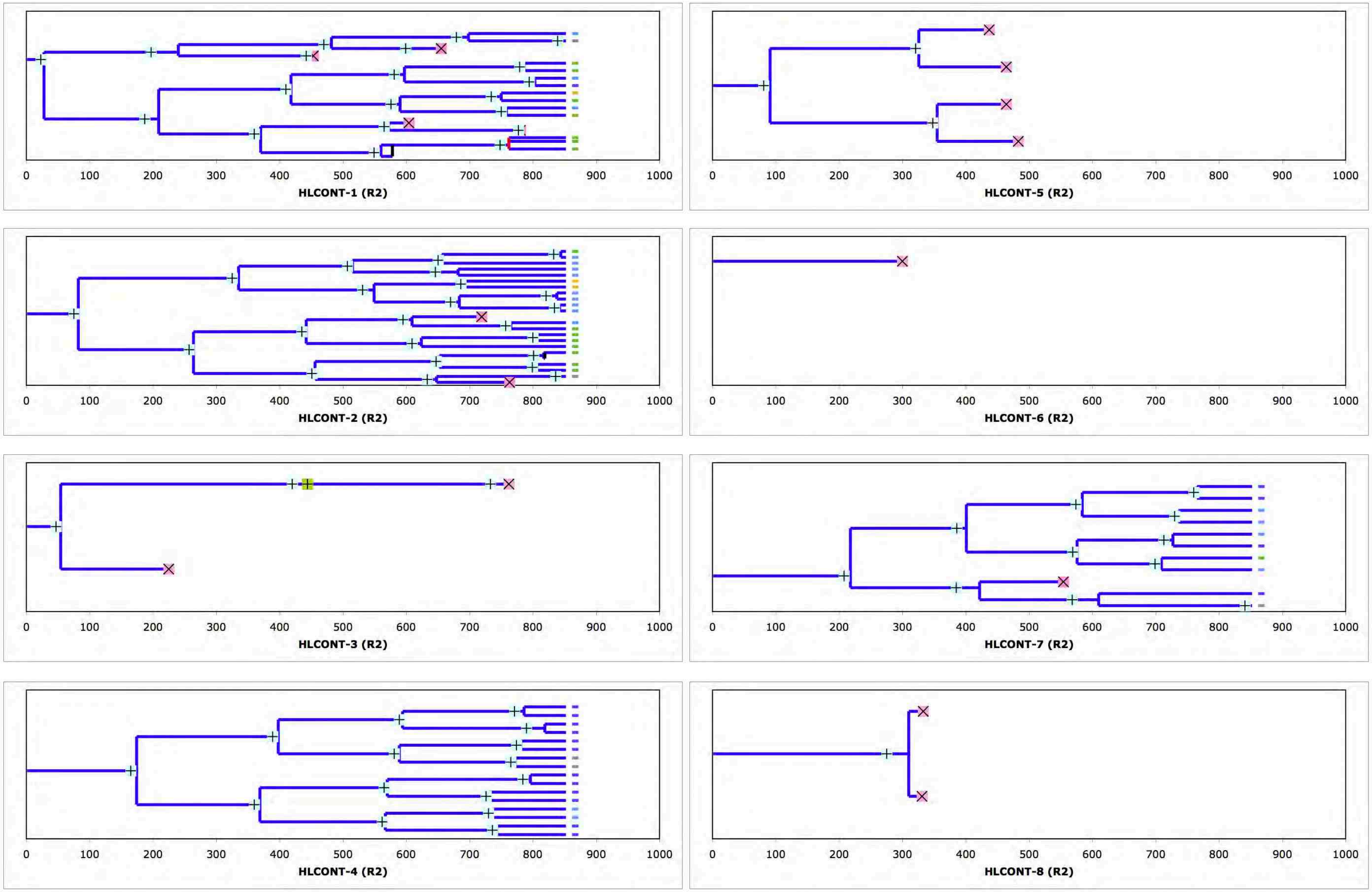

Data Set 5 (HeLa Control R2), Lineage map 9-16

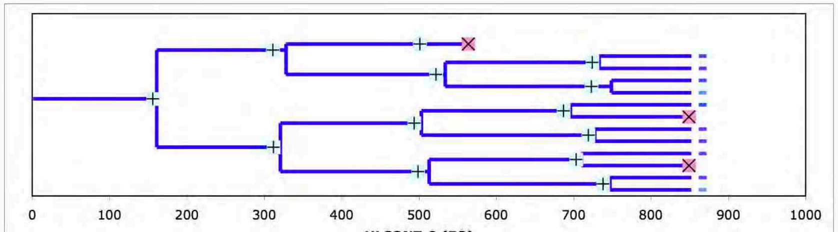

HLCONT-9 (R2)

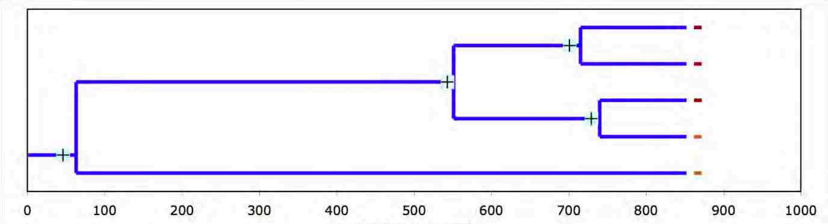

HLCONT-13 (R2)

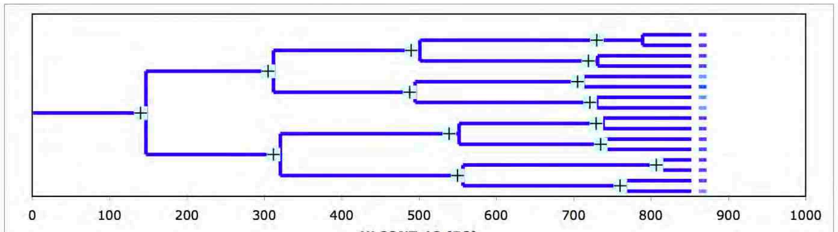

HLCONT-10 (R2)

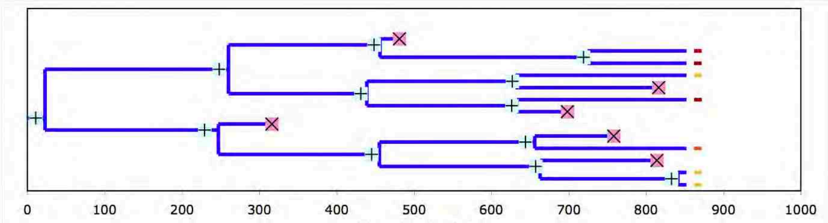

HLCONT-14 (R2)

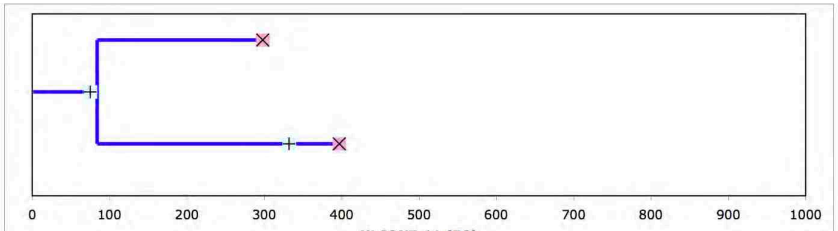

HLCONT-11 (R2)

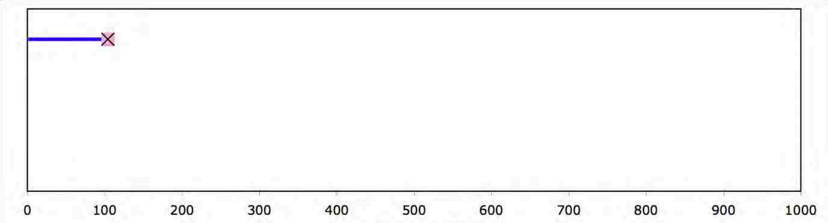

HLCONT-15 (R2)

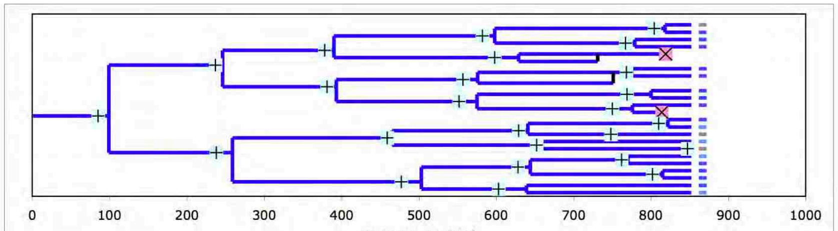

HLCONT-12 (R2)

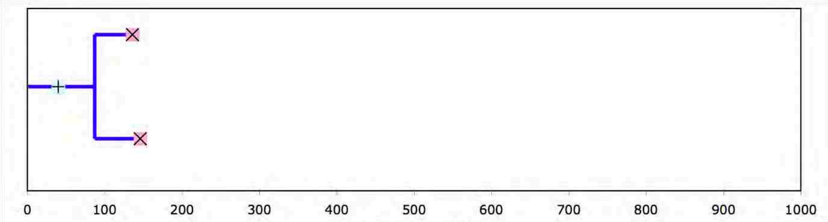

HLCONT-16 (R2)

Data Set 5 (HeLa Control R2), Lineage map 17-24

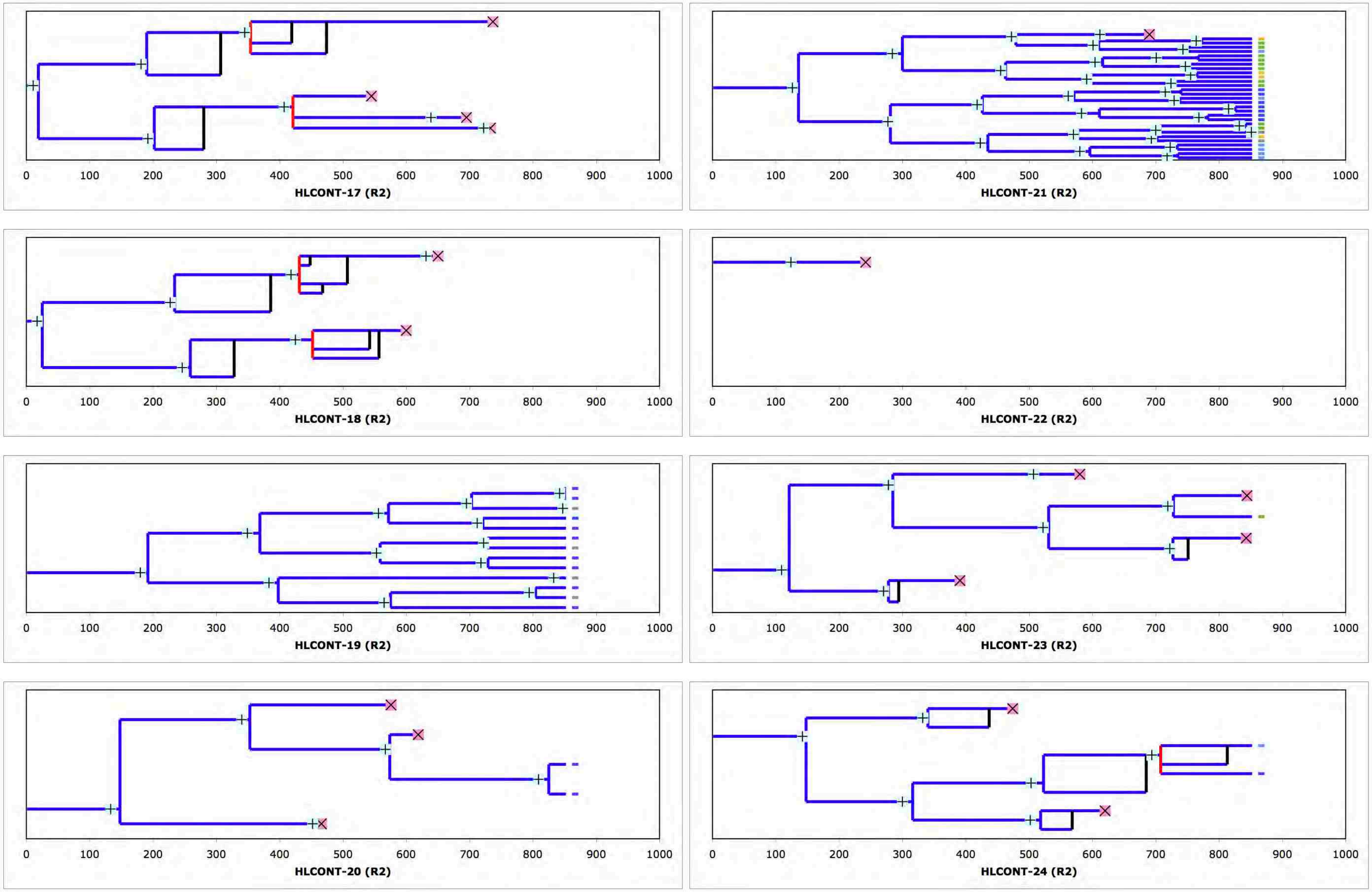

Data Set 5 (HeLa Control R2), Lineage map 25-32

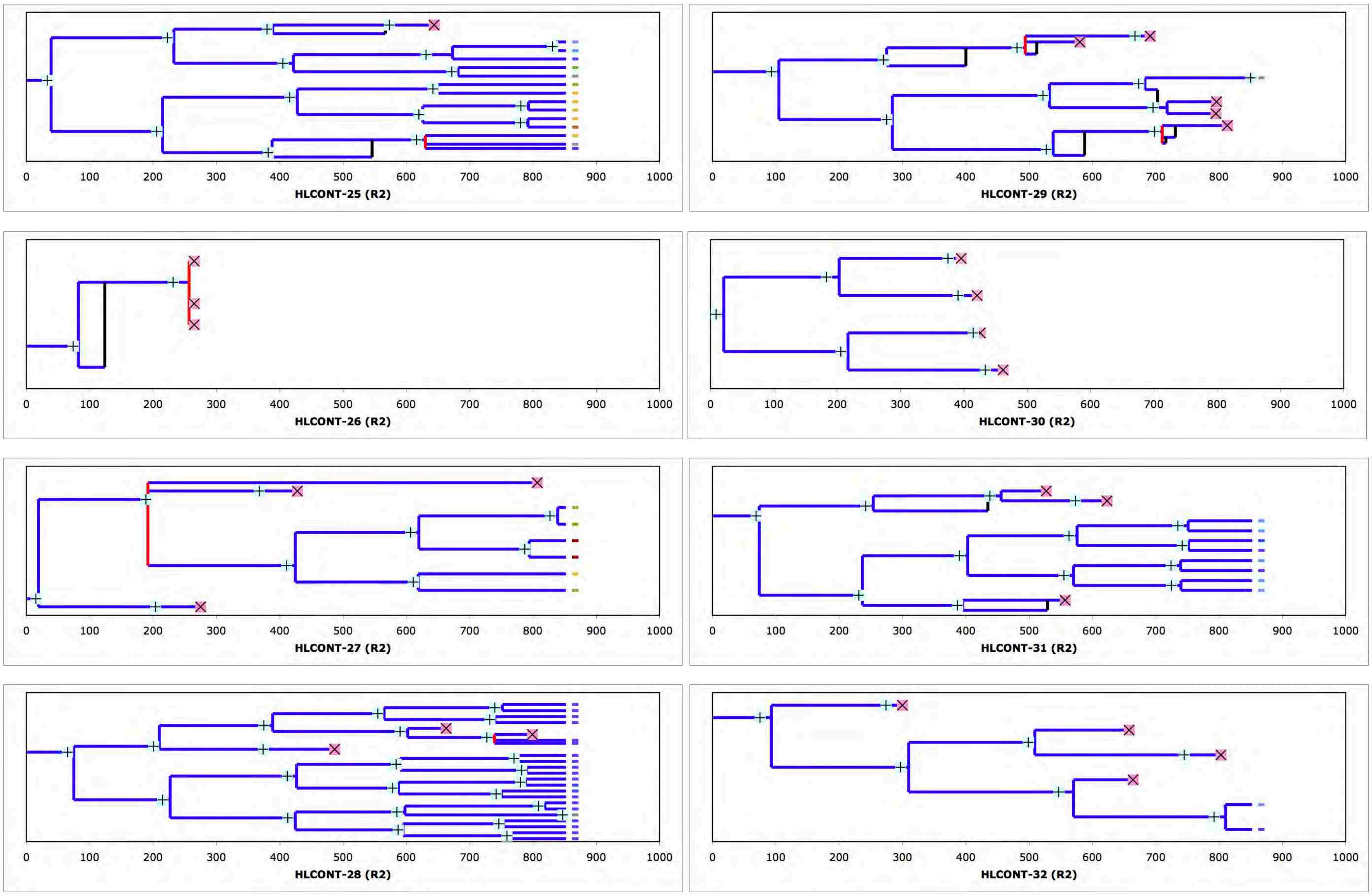

Data Set 5 (HeLa Control R2), Lineage map 33-40

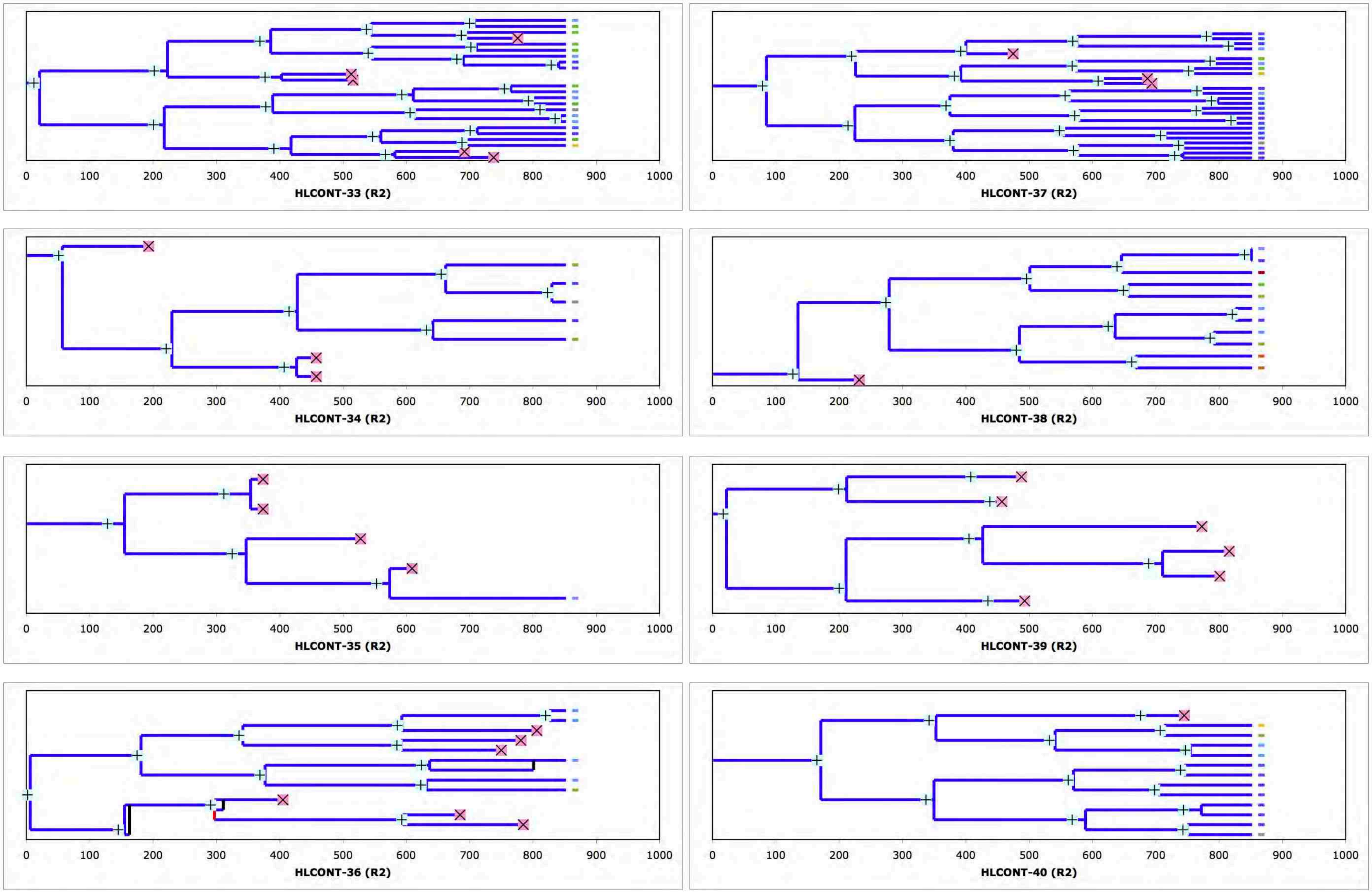

Data Set 5 (HeLa Control R2), Lineage map 41-48

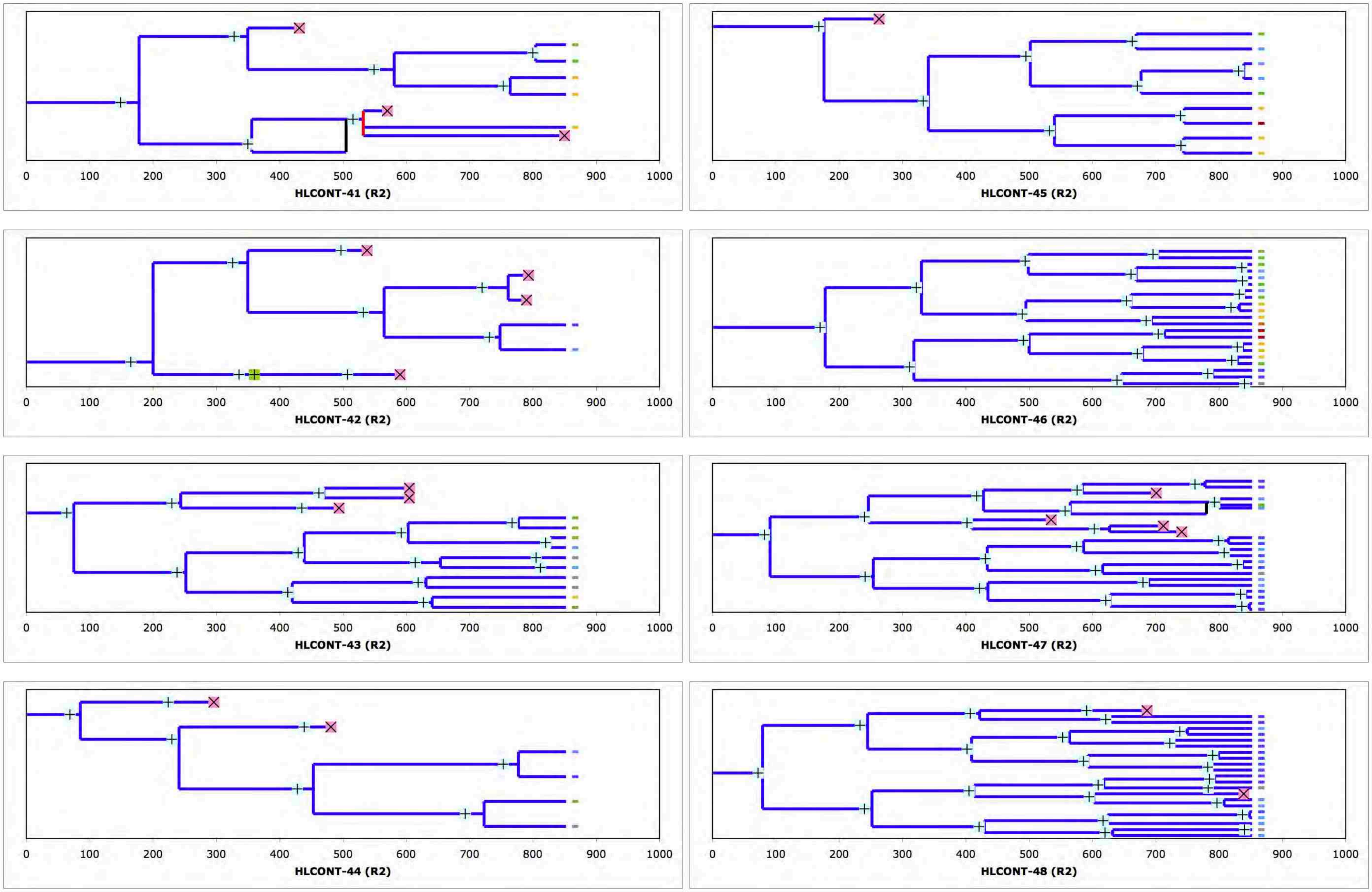

Data Set 5 (HeLa Control R2), Lineage map 49-56

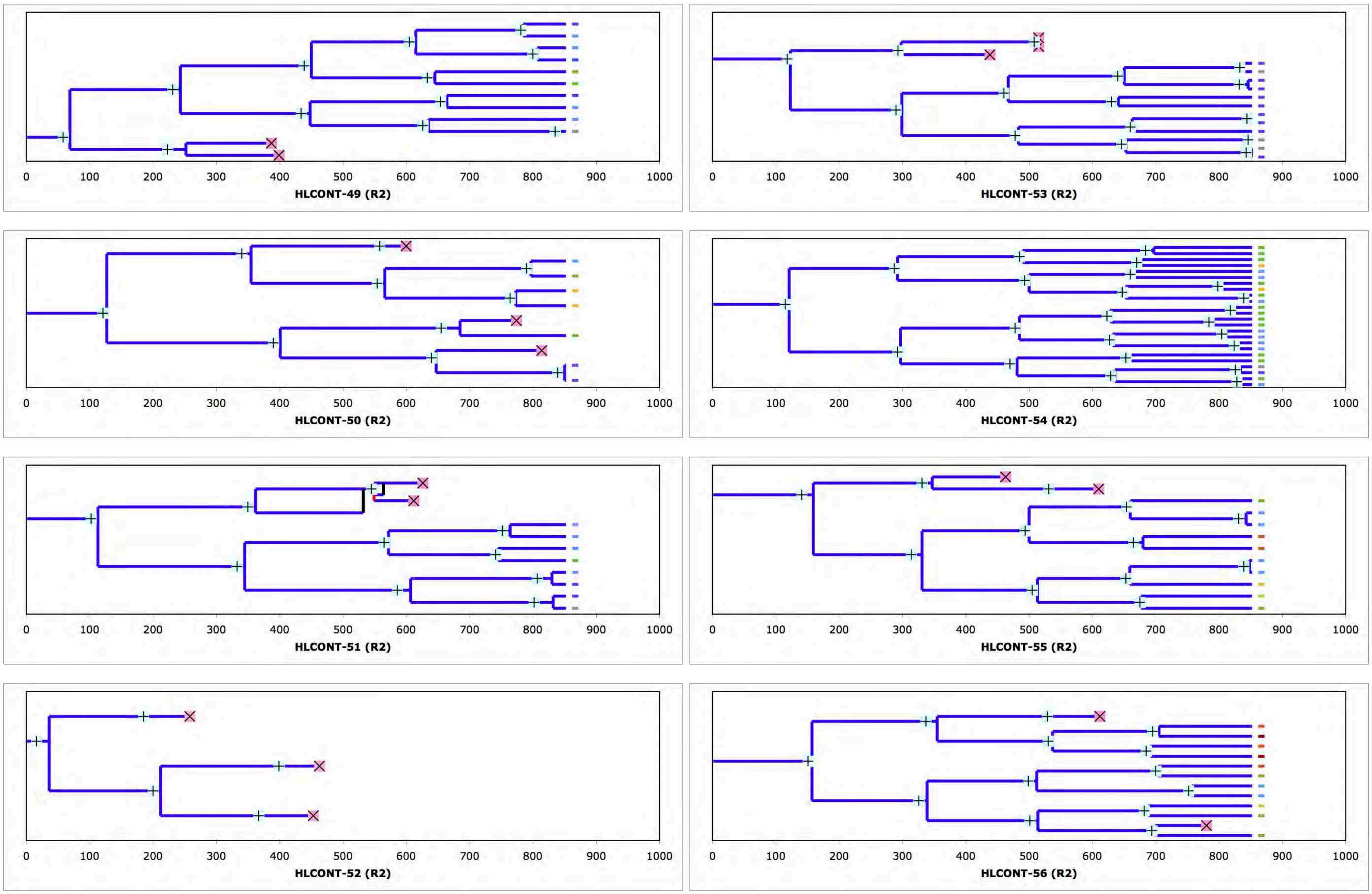

Data Set 5 (HeLa Control R2), Lineage map 57-64

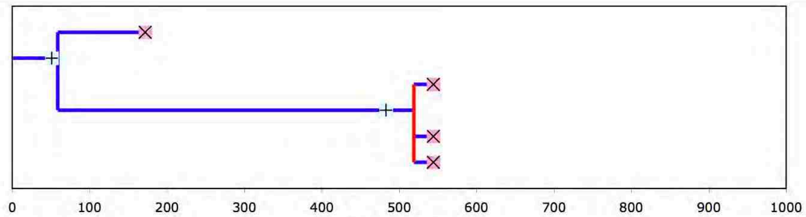

HLCONT-57 (R2)

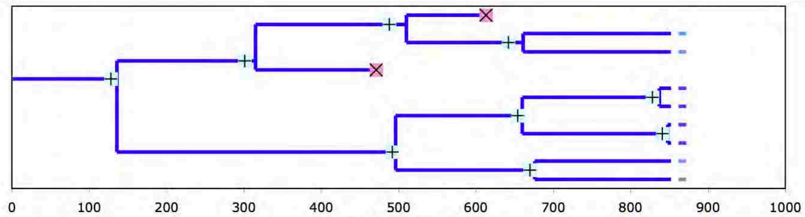

HLCONT-61 (R2)

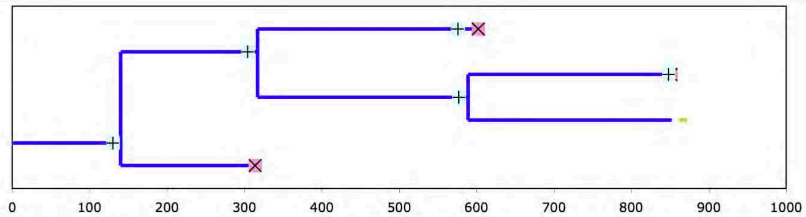

HLCONT-58 (R2)

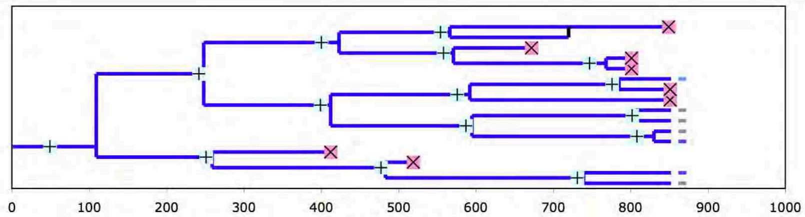

HLCONT-62 (R2)

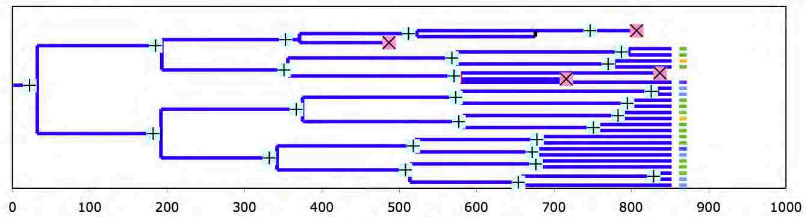

HLCONT-59 (R2)

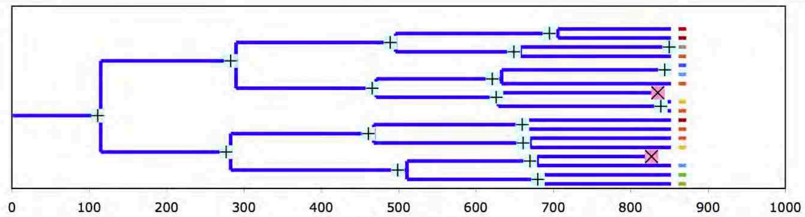

HLCONT-63 (R2)

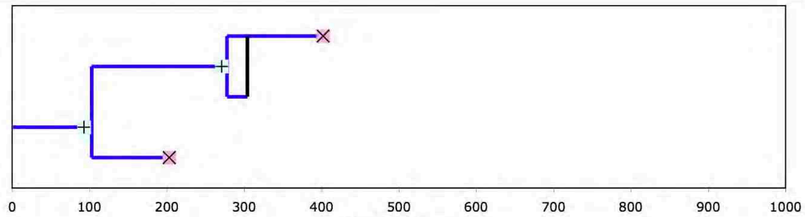

HLCONT-60 (R2)

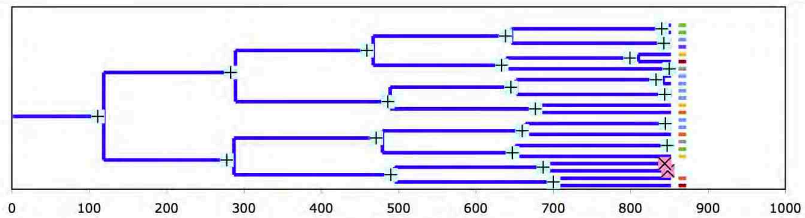

HLCONT-64 (R2)

Data Set 5 (HeLa Control R2), Lineage map 65-72

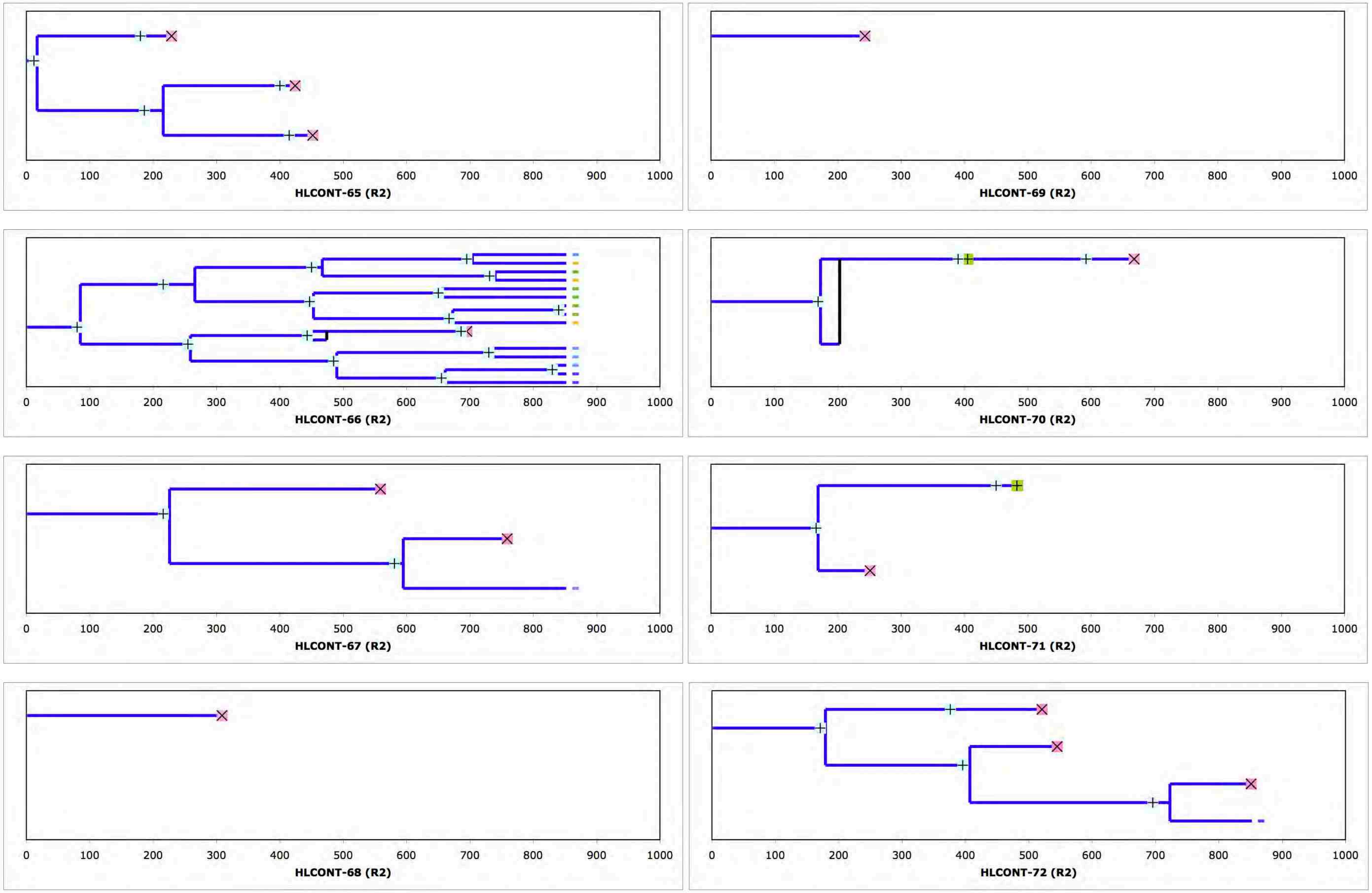

Data Set 5 (HeLa Control R2), Lineage map 73-79

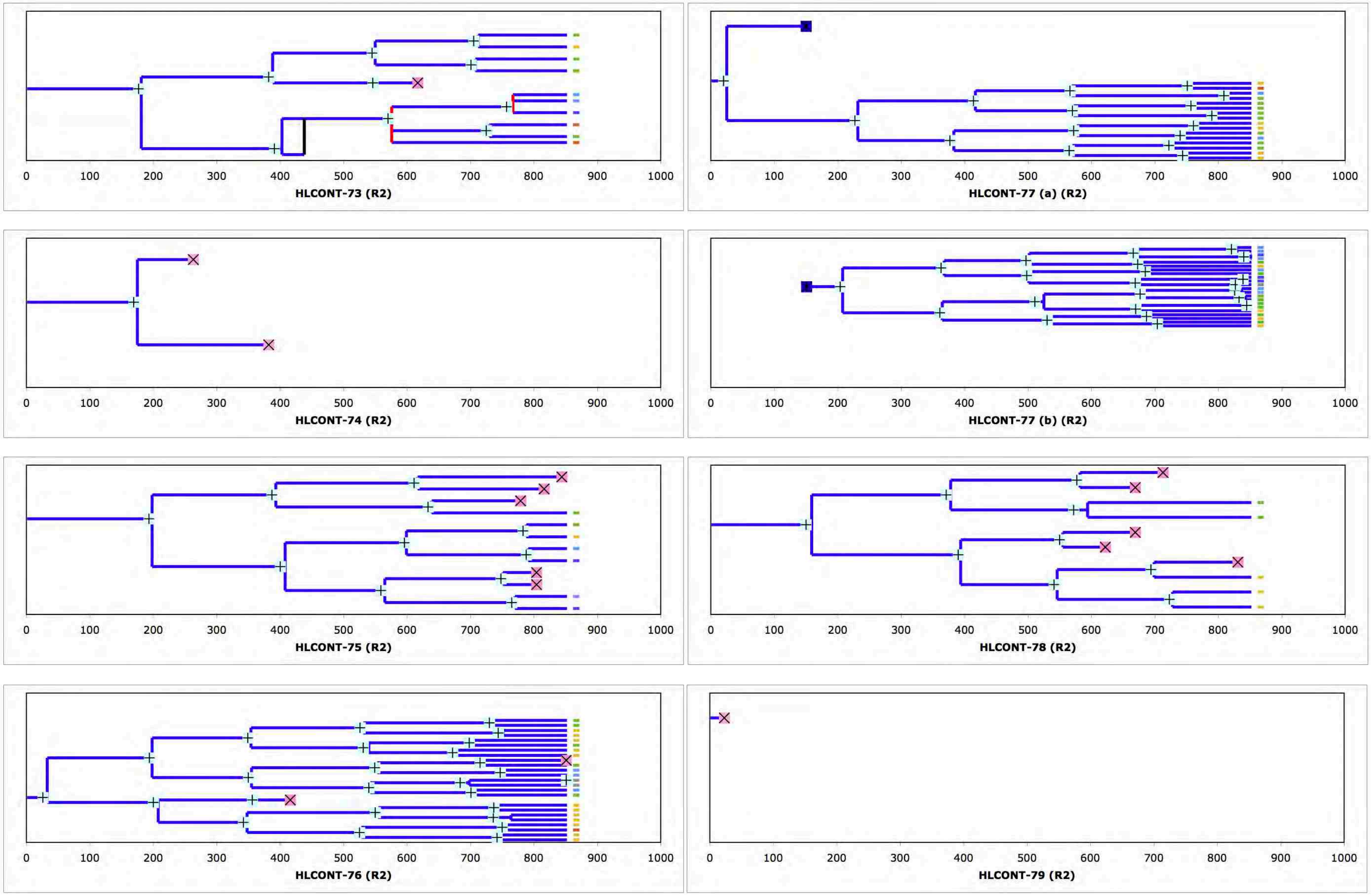

Data Set 5 (HeLa Control R2), Lineage map 80-87

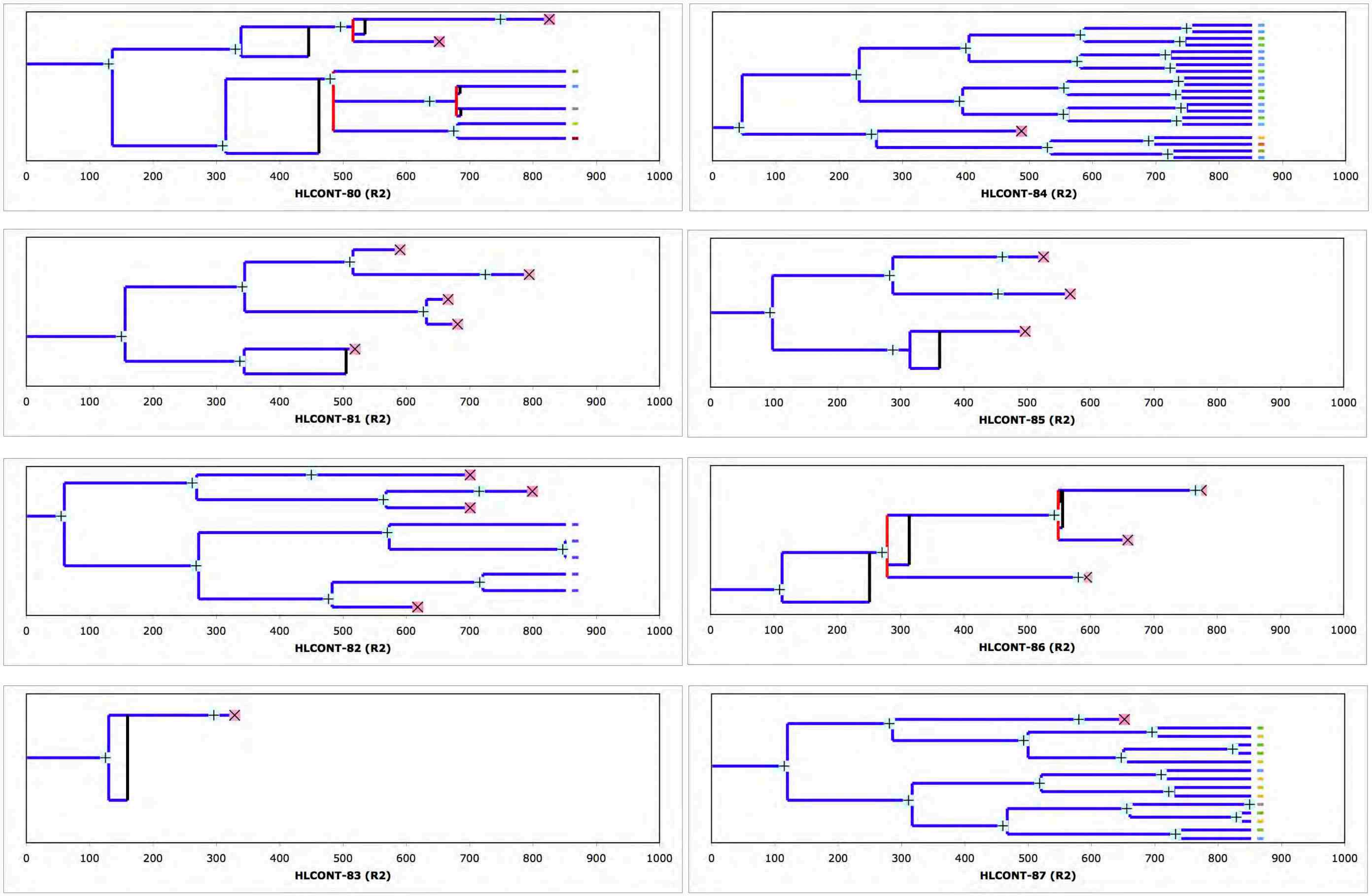

Data Set 5 (HeLa Control R2), Lineage map 88-95

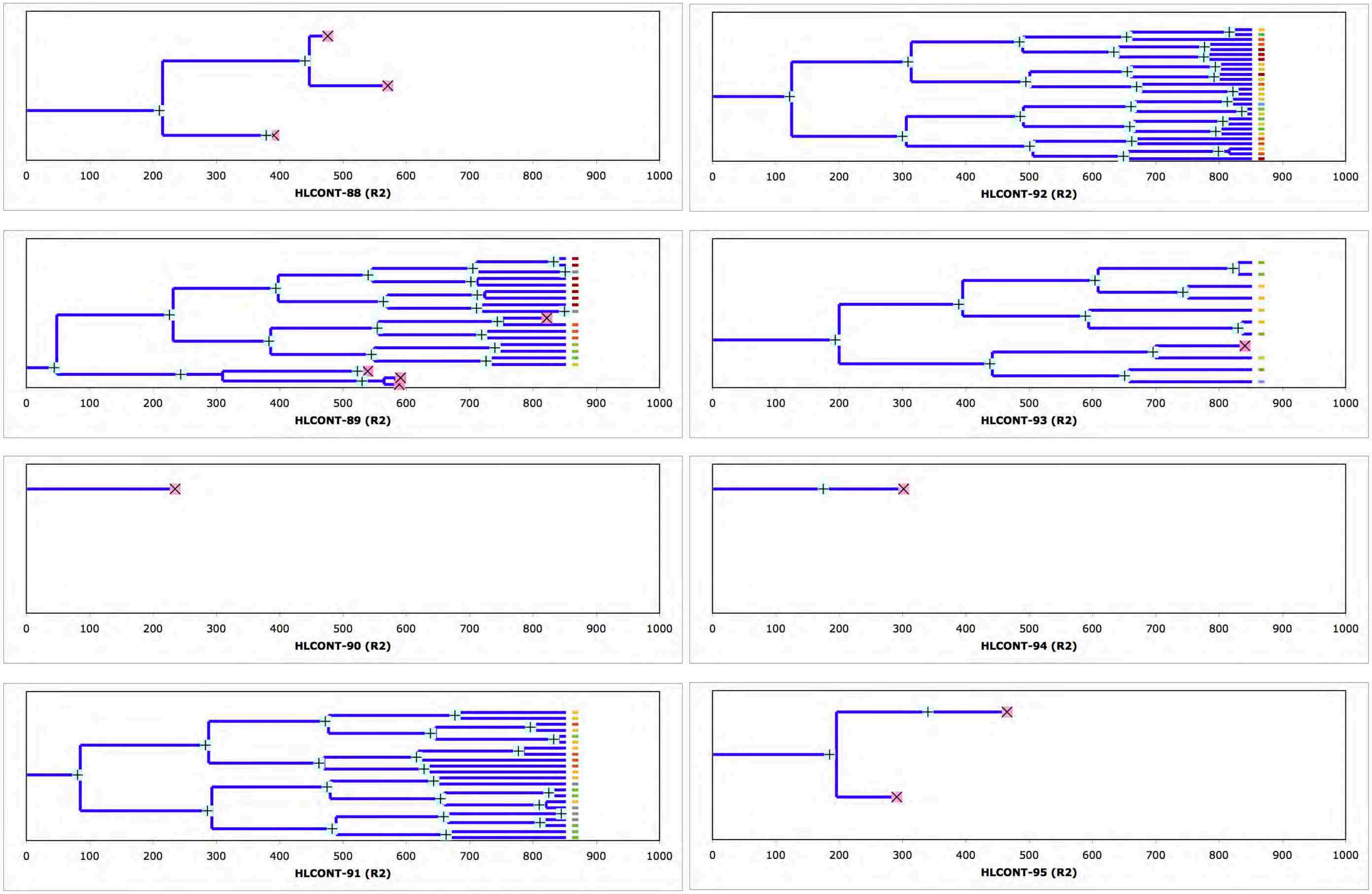

Data Set 5 (HeLa Control R2), Lineage map 96-100

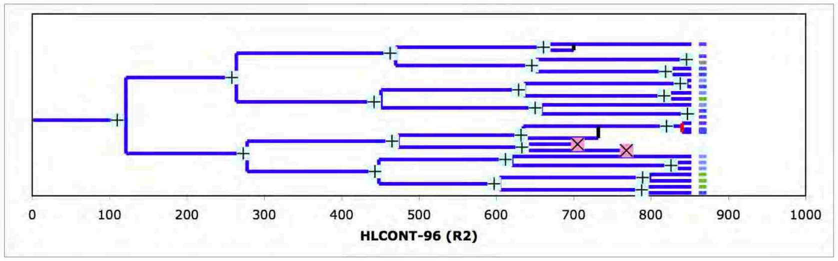

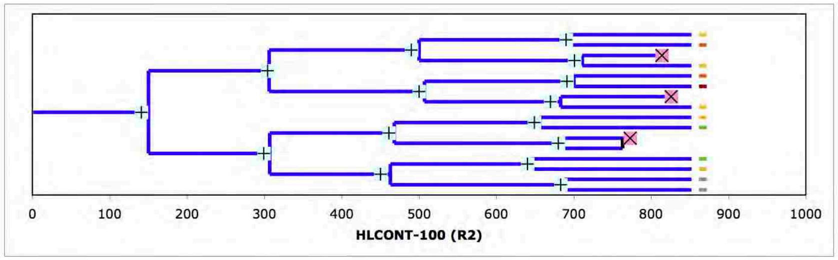

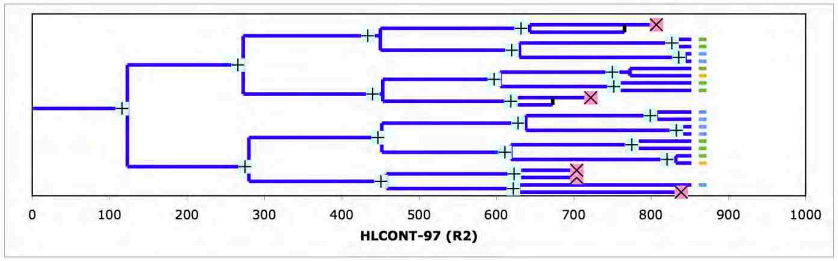

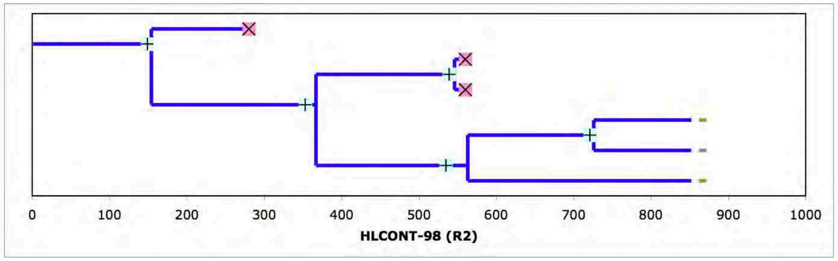

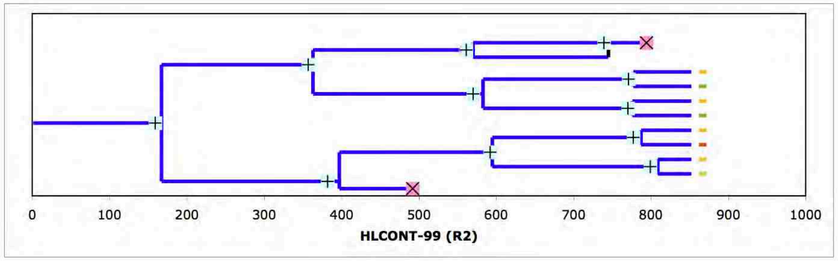

Supplementary Figure S5

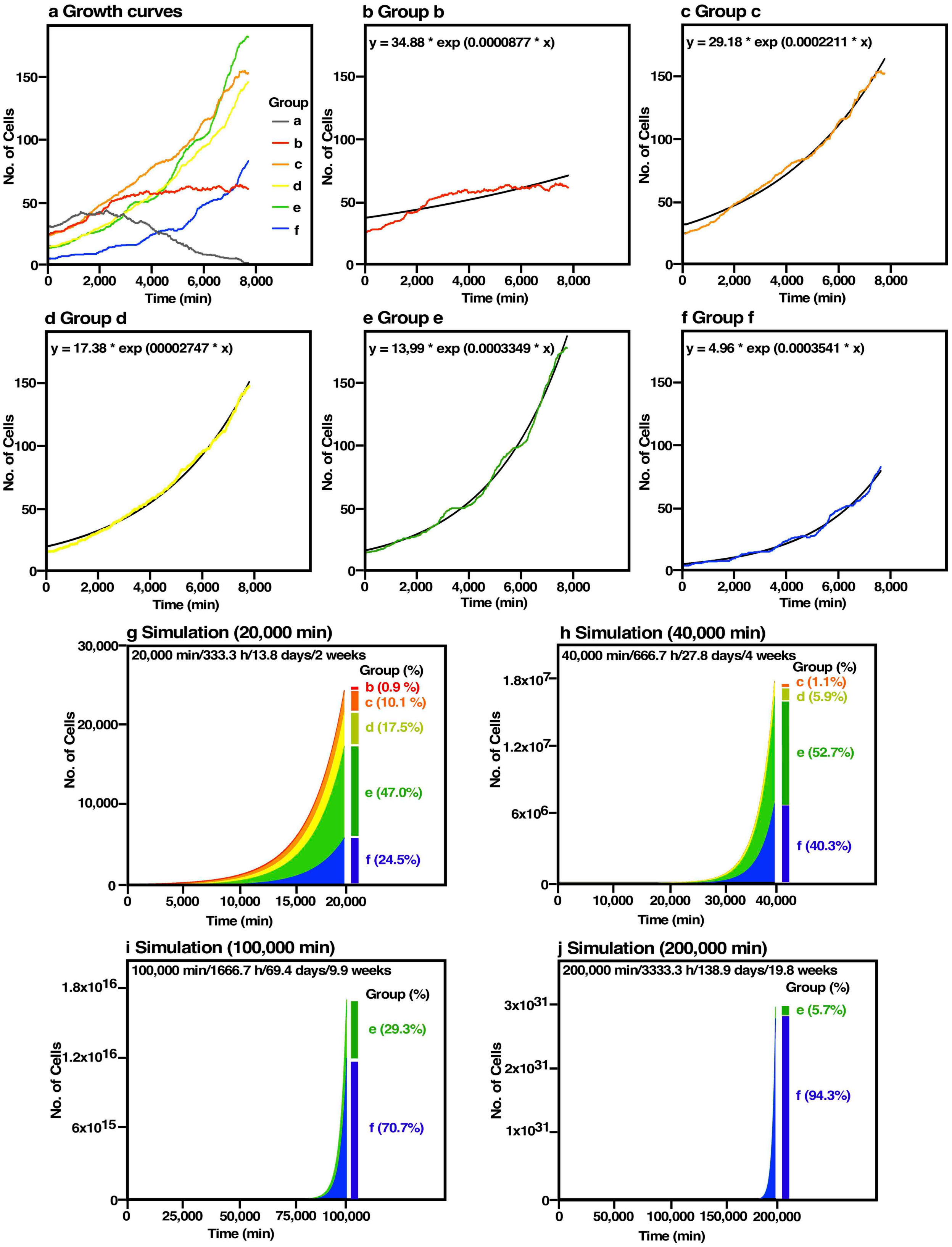

Supplementary Figure S6

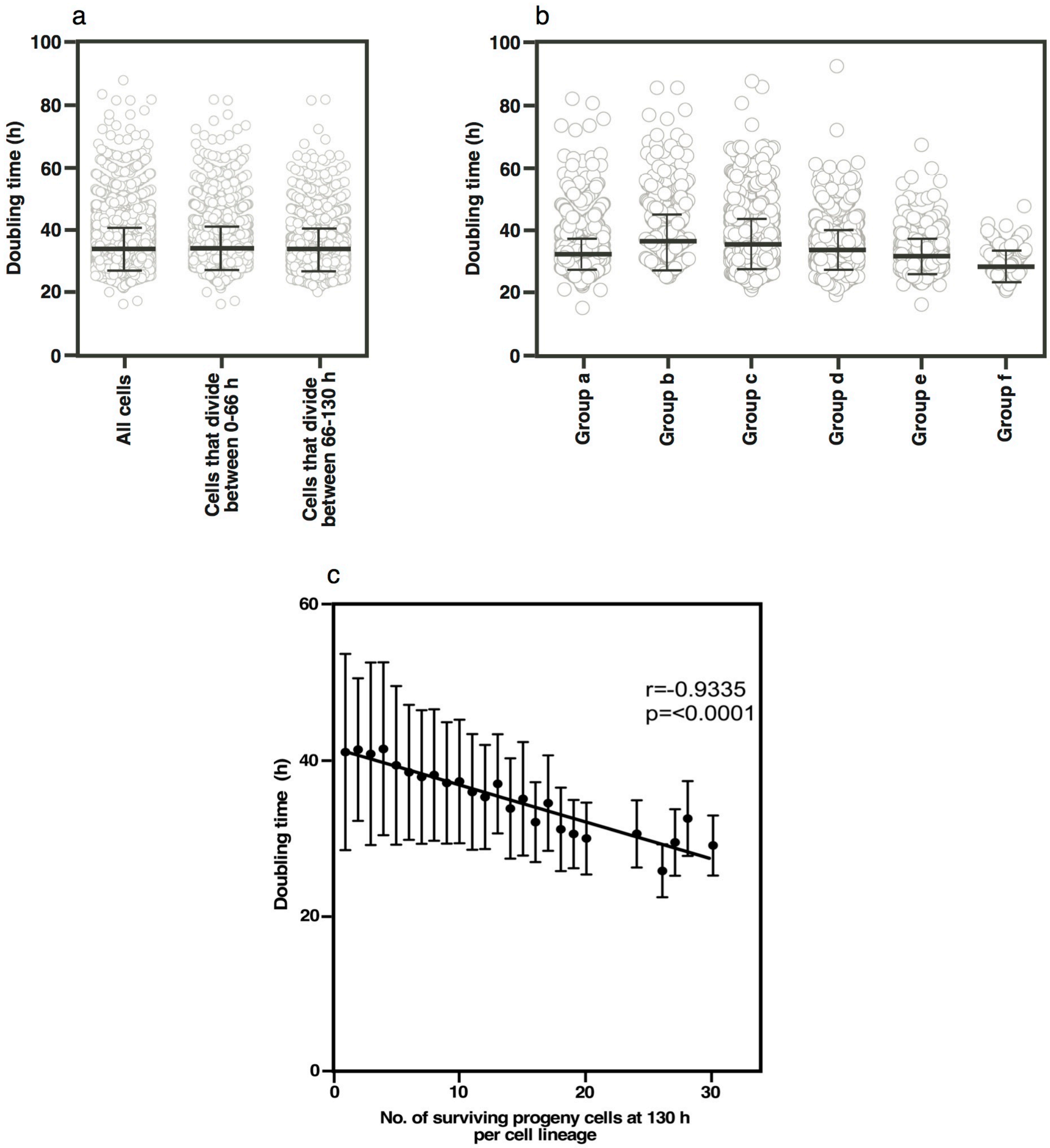

Supplementary Figure S7

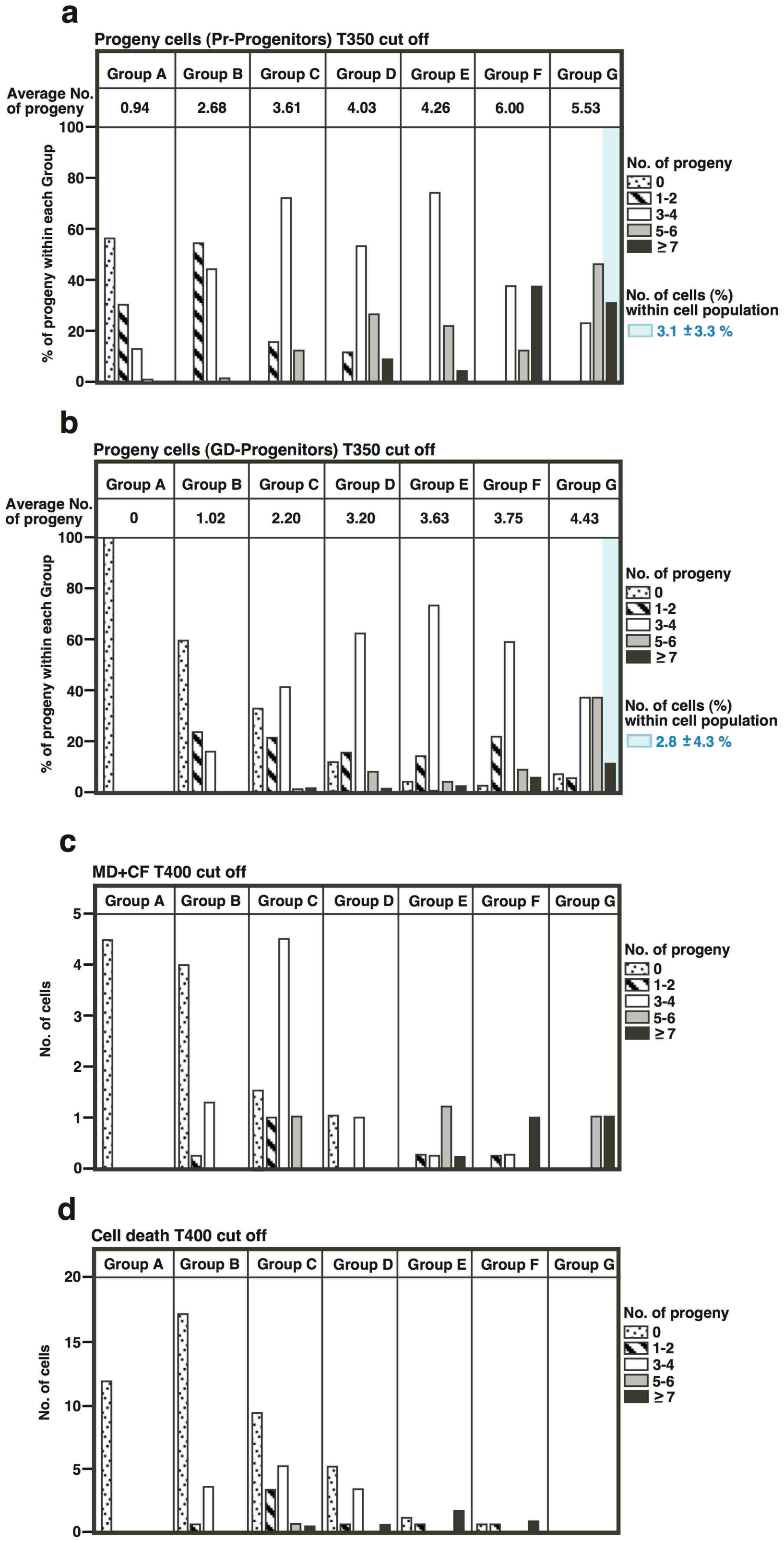

Supplementary Figure S8

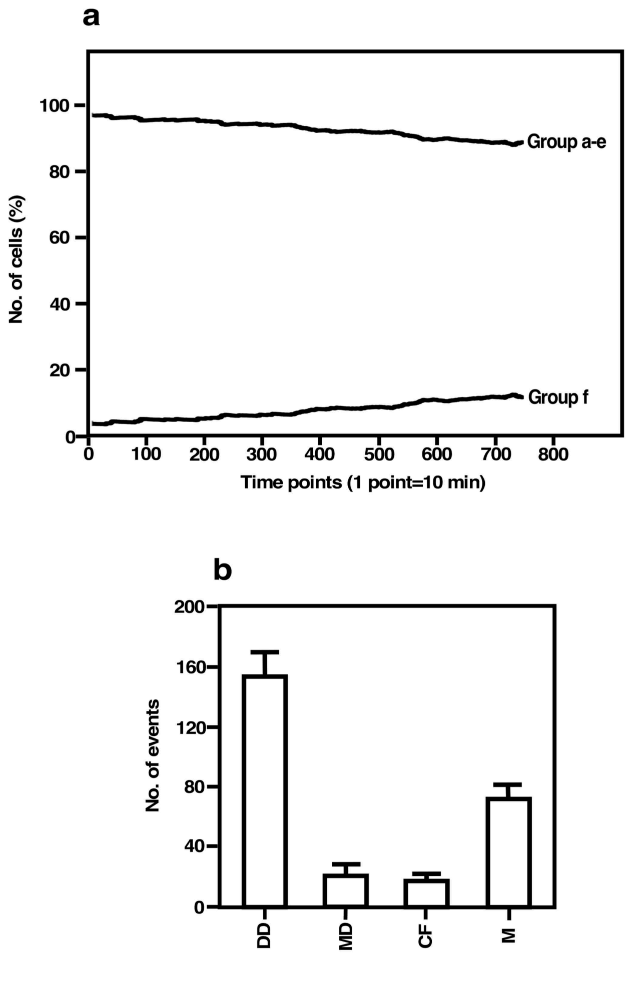

Supplementary Figure S9

a

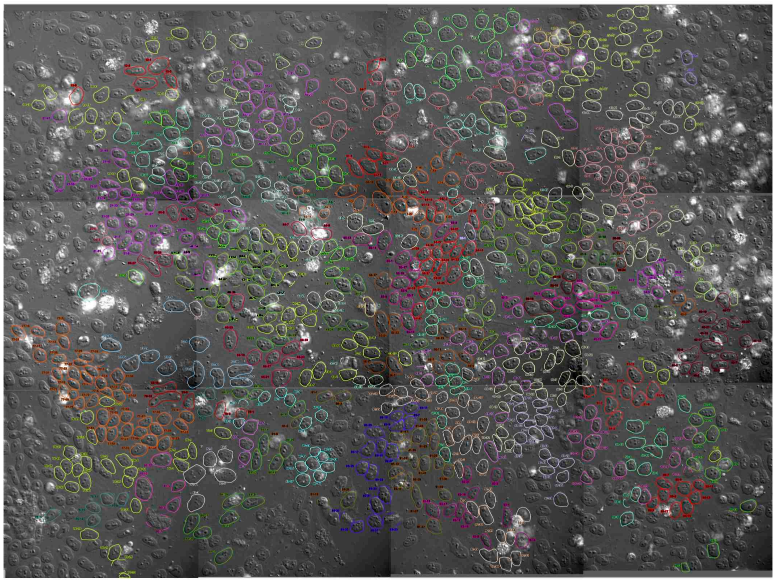

b

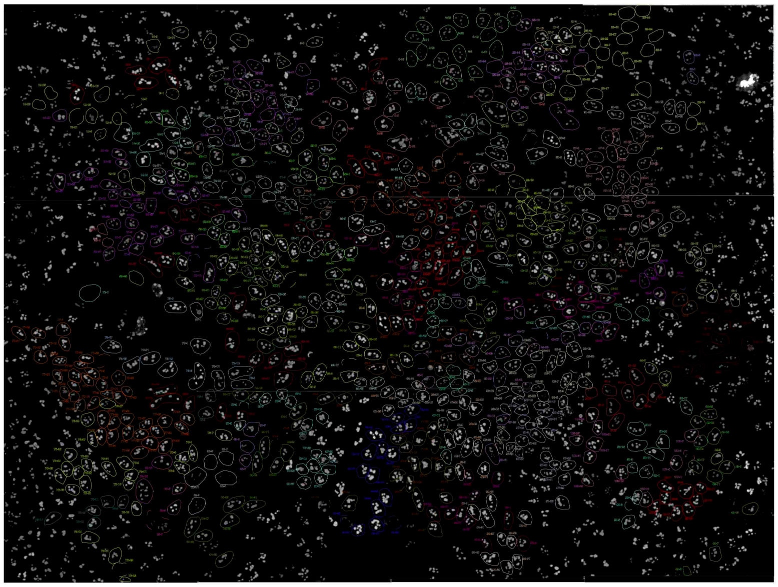

Supplementary Figure S10

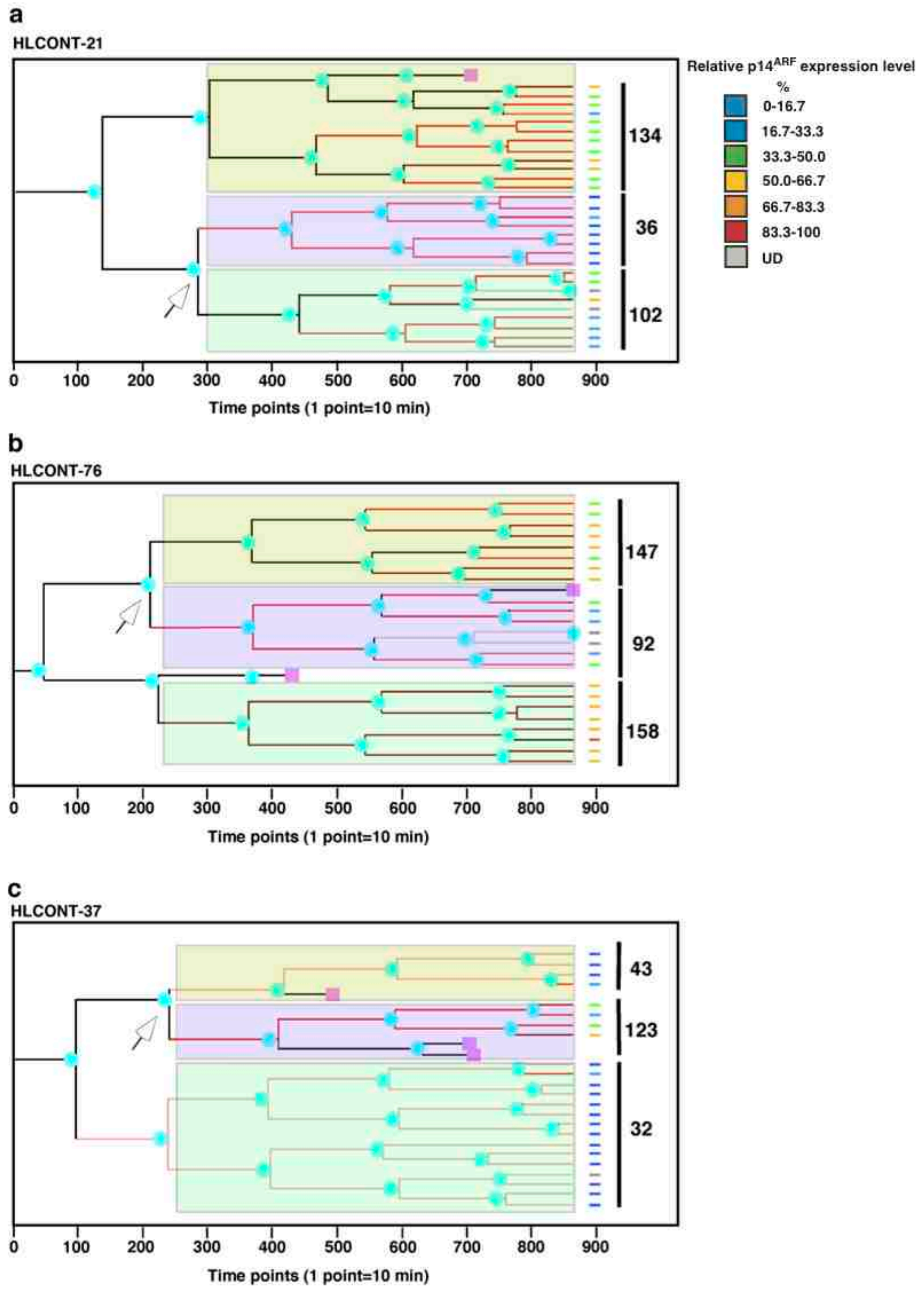

Titles and Legends for Supplementary Movies

Supplementary Movie S1 Growth of HeLa cells on microscope stage
The movie is composed of 963 frames (160.5 h of live cell imaging).

Supplementary Movie S2 Tripolar cell division (TD)
Representative movie of TD is shown. Cell No. C2 underwent TD.

Supplementary Movie S3 Tetrapolar cell division (QD)

Representative movie of QD is shown. Cell No. C17 underwent QD.

Supplementary Movie S4 Pentapolar cell division (PD)

Representative movie of PD is shown. Cell No. C6 underwent PD.

Supplementary Movie S5 Cell fusion between daughter cells
Representative movie of CF is shown. Cell No. C10 underwent DD at T=215. Then, two progenies, C9 and C11, were fused at T=170.

Supplementary Movie S6 Cell fusion between non-mitotic and mitotic cells
Representative movie of cell fusion between non-mitotic and mitotic cells is shown. Cell No. C2 underwent DD at T=317. One progeny, C1, entered into mitosis at T=495 and fused with another progeny, C3, at T=519. The fused cell was then died.

Supplementary Movie S7 Cell death
Representative movie of cell death is shown.
